# Technology-Assisted Physical Activity Interventions for Older People in Their Home-Based Environment: Scoping Review

**DOI:** 10.2196/65746

**Published:** 2025-09-15

**Authors:** Rosemary Dubbeldam, Rafal Stemplewski, Iuliia Pavlova, Magdalena Cyma-Wejchenig, Sunwoo Lee, Patrick Esser, Ellen Bentlage, Veysel Alcan, Özge Selin Çevik, Eleni Epiphaniou, Francesca Gallè, Antoine Langeard, Simone Gafner, Mona Ahmed, Niharika Bandaru, Arzu Erden Güner, Evrim Göz, Ilke Kara, Ayşe Kabuk, Ilayda Türkoglu, Zada Pajalic, Jan Vindiš, Damjan Jaksic, Uǧur Verep, Ioanna Chouvarda, Vera Simovska, Yael Netz, Jana Pelclova

**Affiliations:** 1 Department of Movement Science, Institute for Sport and Exercise Sciences, University Münster, Horstmarer Landweg 62B, Münster, 48149, Germany, 49 15785719390; 2 Department of Digital Technologies in Physical Activity, University of Physical Education, Poznan, Poland; 3 Department of Theory and Methods of Physical Culture, Ivan Boberskyj Lviv State University of Physical Culture, Lviv, Ukraine; 4 Faculty of Physical Culture, Palacký University Olomouc, Olomouc, Czech Republic; 5 Centre for Movement, Occupation and Rehabilitation Sciences, Oxford Brookes University, Oxford, United Kingdom; 6 Department of Electrical and Electronics Engineering, Faculty of Engineering, Tarsus University, Tarsus, Turkey; 7 Department of Physiology, Faculty of Medicine, Mersin University, Mersin, Turkey; 8 School of Humanities, Social and Education Sciences, European University Cyprus, Engomi, Cyprus; 9 Department of Medical, Movement and Wellbeing Sciences, University of Naples Parthenope, Naples, Italy; 10 Normandy University, UNICAEN, INSERM, COMETE, CYCERON, Caen, France; 11 School of Health Sciences, HES-SO Valais-Wallis, Leukerbad, Switzerland; 12 Institut of Sport Science, Otto-Von Guericke Universität of Magdeburg, Magdeburg, Germany; 13 Department of Physiotherapy and Rehabilitation, Faculty of Health Sciences, Karadeniz Technical University, Trabzon, Turkey; 14 Department of Physiotherapy and Rehabilitation, Faculty of Health Sciences, Tarsus University, Tarsus, Turkey; 15 Department of Physical Therapy and Rehabilitation, Institute of Health Sciences, Dokuz Eylul University, Izmir, Turkey; 16 Faculty of Health Sciences, Zonguldak Bülent Ecevit University, Zonguldak, Turkey; 17 Department of Fundamentals of Nursing, Hamidiye Faculty of Nursing, Health Sciences University, İstanbul, Turkey; 18 Faculty of Health and Social Sciences, University of South-Eastern Norway, Kongsberg, Norway; 19 Faculty of Sport and Physical Education, University of Novi Sad, Novi Sad, Serbia; 20 Lab of Computing, Medical Informatics and Biomedical-Imaging Technologies, School of Medicine, Aristotle University of Thessaloniki, Thessaloniki, Greece; 21 HEPA Macedonia National Organisation for the Promotion of Health-Enhancing Physical Activity, Skopje, North Macedonia; 22 The Levinsky-Wingate Academic Center, Wingate Institute, Netanya, Israel

**Keywords:** technology-assisted interventions, technological interventions, physical activity, physical exercise, exercise, gerontology, geriatric, older adult, elderly, older people, aging, home-based, dropout, adherence, adverse effects, scoping review, review

## Abstract

**Background:**

Technology-assisted physical activity interventions for older adults in their home-based environment have been used to promote physical activity. Previous research has reported that such interventions benefit body composition, aerobic fitness, cognitive abilities, and postural control, reducing the risk of falls and maintaining regular physical activity among the older population.

**Objective:**

While previous reviews on technology-assisted physical activity interventions focused on health-related outcomes, this scoping review explores the characteristics of the technology in relation to the characteristics of populations, purpose of the activity, and usability in terms of adverse events, drop-outs, adherence, and user experience.

**Methods:**

A full search was performed in Medline, Embase, CINAHL, SportDiscus, and Web of Science. Sources were considered for inclusion if the participants aged on average 60 years and older, if the physical activity intervention was assisted by technology, and if performed within home-based environments.

**Results:**

We identified 8496 sources. After title and abstract screening, 455 full texts were assessed, and 148 were included, representing 12,717 participants aged 74 (SD 6) years. In total, 63% (93/148) of the sources reported on the population’s health status. The main purpose of the interventions was balance (75/148, 51%), and strength and power (64/148, 43%) and the intervention purposes were not related to the embedded technology. In studies where the participant’s health status was reported as healthy, 53% (78/148) implemented exergames compared to only 27% (40/148) in studies with participants with a clinical condition. Mobile apps (30/148, 20%) and trackers (16/148, 11%) were implemented likewise in both groups. The technology was embedded to provide continuous exercise information (40/148, 27%) and exercise feedback (40/148, 27%) or to record real-time movement data (38/148, 26%). Adverse events were reported in 46% (68/148) of the sources with three quarters (49/68) reporting no adverse events. Only two mild events were related to technology. Dropout rates were reported in 68% (100/148) of the sources, with no differences between intervention (16 SD 16%) and control (14 SD 12%) groups. Dropout reasons related to technology are only 3% (3/100). Adherence was reported in 53% (79/148) sources and was slightly higher in the intervention group (80 SD 18%) compared to the control group (71 SD 25%). A significantly higher adherence was found between interventions that were tailored (83 SD 15%) versus those that were not (75 SD 21%). General enjoyment of the technology was captured in 37% (55/148) of the sources. Within those sources, 91% rated positive (91/100), 7% neutral (7/100), and 2% negative (2/100). Occasionally reported wishes were related to goal setting, feedback, technical support, exercise variation, and social setting.

**Conclusions:**

Various technologies were successfully used in healthy and clinical older populations, though evidence regarding their implementation in physical activity interventions in hospital settings remains limited. The embedded technology was not a reason for additional dropouts, led to slightly better adherence, and adverse events were rarely related to technology. When assessed, the technology was well accepted and positively enjoyed.

## Introduction

The benefits of physical activity in advanced age are well-documented, yet paradoxically, sedentary behavior increases with age [1,2]. Even if individuals are motivated to engage in physical activity outside the home, environmental barriers such as weather conditions, infrastructure constraints, or specific constraints such as COVID-19 can pose challenges for older adults [3,4]. This paradox has prompted clinicians to seek technological solutions to encourage older adults to be more physically active within their home environment. It is important to distinguish between physical activity and exercise. Physical activity encompasses any bodily movement that expends energy, while exercise is a structured subset of physical activity specifically designed to improve fitness components [5].

Various electronic devices, software, or wearable technology such as smartphones [6], fitness trackers [7], mobile phone applications [8], tablets [9], virtual reality [10], exergames [11], E-coaching [12], and Robotics [13] have been reported to support beneficial changes in sedentary time and physical activity in the older adult population [14,15]. Such technological interventions benefit body composition, aerobic fitness, cognitive abilities, and postural control and reduce the risk of falls among the older population [9,16,17]. However, while there are indications that older adults tend to show high levels of adherence to technology-assisted physical activity interventions [15], quite a few barriers limiting the use of this technology have been reported, such as functional barriers related to the design of e-health programs and their interface with older end users, or lack of technological support [18].

With the continuously increasing number of technology-assisted healthcare tools, particularly those for home-based physical activity, and the difficulties older adults face in adapting to and operating these tools, it is not surprising that several reviews have been conducted in recent years to examine the usability of technology-assisted physical activity programs [15,19-25], more than half of them in recent years – 2021‐2024 [19,20,22,23,25]. Most of these reviews focused on a specific population, egfor example, healthy, frail, or people with cardiovascular disease, focused on specific aspects of interventions, for example, balance or falls, or focused on a specific type of technology, for example, exergames [15,19-25]. Considering the boost of assistive-technology provision of physical activity in the recent five years, some are relatively dated [15,24]. A recent review by Costa-Brito et al elaborated on the usage of technology to improve physical function among older community-dwelling adults [23] and focused on the purpose and volume of an activity, types of technology, aspects of the usage of technology (adherence, acceptance, feasibility), and health outcomes. The review, however, did not go into the specific characteristics of the study participants, the technology’s functionality, or its user interface. Furthermore, it did not explore or analyze key aspects of technology design, function, and interface in relation to specific populations or physical activity goals. Just as many other reviews [19-21,25], Costa-Brito et al also neglected to examine safety concerns or adverse events associated with the use of technology.

Aligned with the recommendations of the PhysAgeNet, a European Cooperation in Science and Technology (COST) network devoted to evidence-based physical activity in old age [26] and with the proliferation of technology related to home-based physical activity, our aim in this study was to explore the characteristics of that technology in relation to the characteristics of older populations (eg, age group or health status) and the interventions. Furthermore, we aimed to examine the usability of that technology in terms of adverse events, drop-outs, adherence, and the user experience (acceptability and enjoyment). This may promote the optimal utilization of technology to provide efficient home-based physical activity programs for older adults. A scoping review was selected as the most suitable methodology due to its capacity to encompass a broad spectrum of study designs and methodologies. This approach facilitates the mapping of key concepts and the identification of research gaps, which is particularly critical in this emerging field characterized by diverse evidence sources, including randomized controlled trials, pre-post studies, feasibility studies, qualitative research, and mixed methods investigations.

## Methods

### Review Process

This scoping review was conducted in accordance with the Joanna Briggs Institute (JBI) methodology for scoping reviews [26] and the Preferred Reporting Items for Systematic Reviews and Meta-analyses extension for scoping reviews (PRISMA-ScR) checklist [27]. The protocol for the scoping review is available at the Open Science Framework Registries [28]. The review was conducted by an international interdisciplinary team of researchers with regular weekly meetings to discuss, distribute tasks, and ensure consistency during the different steps of the process.

### Eligibility

Sources were considered for inclusion if they met the following criteria: (1) participants aged on average 60 years and older with and without health conditions; (2) studies that focused on technology-assisted physical activity interventions; and (3) the intervention was based within home-based environments. Table 1 gives a detailed definition of the participants, concept, and context.

**Table 1.** Details of the participants, concept, and context (PCC framework) which form the basis for source inclusion consideration.

PCC	Inclusion	Definitions and descriptions
Population	Older people	Older adults aged 60 years and older (the participant’s group mean age should be ≥60 y), living independently or in residential facilities (aged care facilities), with or without health conditions.
Concept	Technology-assisted physical activity interventions	A physical intervention is a structured or unstructured measure taken to improve or maintain the user’s physical activity and practiced individually. It can be tailored to specific health or fitness goals (structured) or it can be general and not specifically prescribed by a health professional (unstructured) [29]. The intervention includes any form of physical activity such as human daily activities, exercise, training, or physical fitness training. The technologies used to deliver the intervention are digital systems and include, among others, mobile applications, telemedicine, wearable electronic devices, exergaming, and virtual reality. The delivery of the intervention can take place supervised, facilitated (partly supervised), or unsupervised.
Context	Home-based environment	The home-based location and environment, ie, the immediate vicinity in the community environment, ie, a public open-access setting [29]. The participants may be living in a community setting on their own (community dwelling) or in an institution such as a care home or a hospital.
	Health status	People with or without mental or physical impairments.

Sources were excluded if (1) the study design was a review or study protocol, or if the publication type was an abstract or non-peer-reviewed conference proceedings, (2) participants were on average younger than 60 years of age, (3) no physical activity intervention was present or the intervention took place within clinical setting, (4) digital technology was not present or not an integral part of the intervention, (5) the intervention did not take place in home-based setting, or (6) the study language was other than English.

### Search Strategy

According to JBI recommendation, an initial limited search of PubMed and Web of Science was conducted to identify potentially relevant key search terms for the PCC framework. In an iterative process, the keywords in the text of retrieved sources were analyzed. The search terms provided were consulted with experts within the team to capture all relevant keywords and then grouped into four main headings: population group, residence, technology, and intervention. The search string was further expanded by running searches with Medical Subject Heading (MeSH Terms) as well as with adequate non-MeSH terms for databases that do (eg, PubMed) or do not use a MeSH tree (eg, Web of Science), respectively and cross-checked by a subject librarian at Oxford Brookes University: (“aged” OR “elder*” OR “old” OR “older people” OR “geriatric” OR “senior#”) AND (“home*” OR “resident*” OR “care facilit*” OR “nursing home*”) AND (“digital technolog*” OR “mobile health” OR “telehealth” OR “eHealth” OR “mHealth” OR “exergam*” OR “video gam*” OR “serious gam*” OR “wearable technolog*” OR “wearable electronic device*” OR “application*” OR “mobile app*” OR “virtual reality”) AND (“exercise#” OR “training” OR “intervention*” OR “physical activit*” OR “physical fitness”) NOT (“review”[Title/Abstract] OR “rehabilitation” [Title/Abstract])

The created search strings were the basis for developing a full search strategy in Medline (PubMed), Embase (Ovid), CINAHL (Ebsco), SportDiscus (Ebsco), and Web of Science and converted as necessary (Multimedia Appendix 1). The search in the different databases was performed between September 26 and October 6, 2022, and all sources published in previous years were included. As a third step of the search strategy, an additional search was performed in the reference list of the identified sources selected from the full text search.

### Source of Evidence Selection

Following the search, all identified sources were collated and uploaded into Rayyan [30], and duplicates were removed. Two reviewers performed a pre-screening pilot on 100 titles and abstracts to develop screening instructions (total agreement=95%, Cohen’s Kappa=0.701). Any disagreements were resolved via consensus with the help of a third reviewer. After the whole team approved the final version of the screening instruction tool, the 2 reviewers screened the remaining titles and abstracts (total agreement=95.5%, Cohen’s Kappa=0.515). Again, disagreements were resolved by a third reviewer.

Full texts of potentially eligible sources were uploaded into Rayyan. Ten different pairs of reviewers assessed these sources (total agreement =81%, Cohen’s Kappa=0.696) within Rayyan [30]. Disagreements were resolved via consensus in a team of five other (independent) reviewers. The results of the search, the sources’ inclusion process, and exclusion reasons have been reported in full and presented according to the PRISMA flow diagram [27]. All sources obtained after the search process were stored in Mendeley (Mendeley Ltd., Elsevier).

### Data Extraction

A data extraction tool (Multimedia Appendix 2) was made in line with the JBI guidelines [26,31] and a template for intervention description and replication [32]. Five reviewers piloted the draft extraction tool, topic explanations were added to ensure the correct data was extracted into relevant columns and a final data extraction tool was prepared after which a group analysis and discussion was conducted, making sure that each extractor understood the tool. Finally, ten pairs of reviewers independently completed data extractions. When preparing the data for the analysis, a third reviewer cross-checked the extracted data and, where necessary, consulted the full text for details. Discrepancies and uncertainties were discussed within the team.

### Data Analysis and Presentation

Four teams consisting of 3‐5 reviewers analyzed the extracted data regarding the following topics: study design, participants, interventions and outcome measures, and technologies and key findings. An inductive thematic analysis was conducted. Codes or labels were defined to categorize the data and a coding framework with definitions of the categories and subcategories was developed. This coding framework allowed us to characterize the participants (age, health status, mobility, country of residence, etc), intervention (purpose, tailoring, temporal data, etc), the used technologies (design, function, interface), participation (feasibility, effectiveness, safety, etc), and health outcomes (physical and mental capacities, fall risk, quality of life, etc). A detailed description of the coding framework is presented in Multimedia Appendix 3. During the extracted data categorization process, the reviewers came together regularly in their small teams to discuss discrepancies until consensus was achieved and all data categorized. For this review, the focus lies with the participants, intervention, and especially the technology categories.

The quantitative and qualitative data were analyzed descriptively using absolute and relative frequency, percentage, average, and standard deviation. Pie charts and bar charts were made to visualize results.

Crosstabulation statistical analysis was used to get a better understanding of what kind of digital technologies were used to integrate specific physical activity intervention programs and for which populations. Observations between 2 or more nominal variables were explored, whereby the frequency of observations between variables was counted and reported accordingly and expressed as a percentage against all observations. Specifically, the cross-tabulation analysis was conducted between the following categorical variables: Technology (design, function, interface) versus Population (age group, health status, residency) and Intervention (aim of physical activity, tailoring).

Adherence (attendance and duration) and dropout rates were expressed as a percentage of the relevant study population and compared against population and intervention characteristics (2 or more categories) using differential tests. In the case of a binomial category, an independent *t* test was used. The following categories and linear outcomes were compared: Participation outcomes (dropouts, adherence) versus Population (age groups, health status) and Intervention (group, tailoring, supervision).

Relationships between linear parameters were regressed to extract the coefficient of determination (*R*^2^) with associated *P* value to indicate its significance. These were tested for the dropout and adherence outcomes, against the total number of intervention sessions and session durations.

## Results

### Source Selection

**Figure 1.**
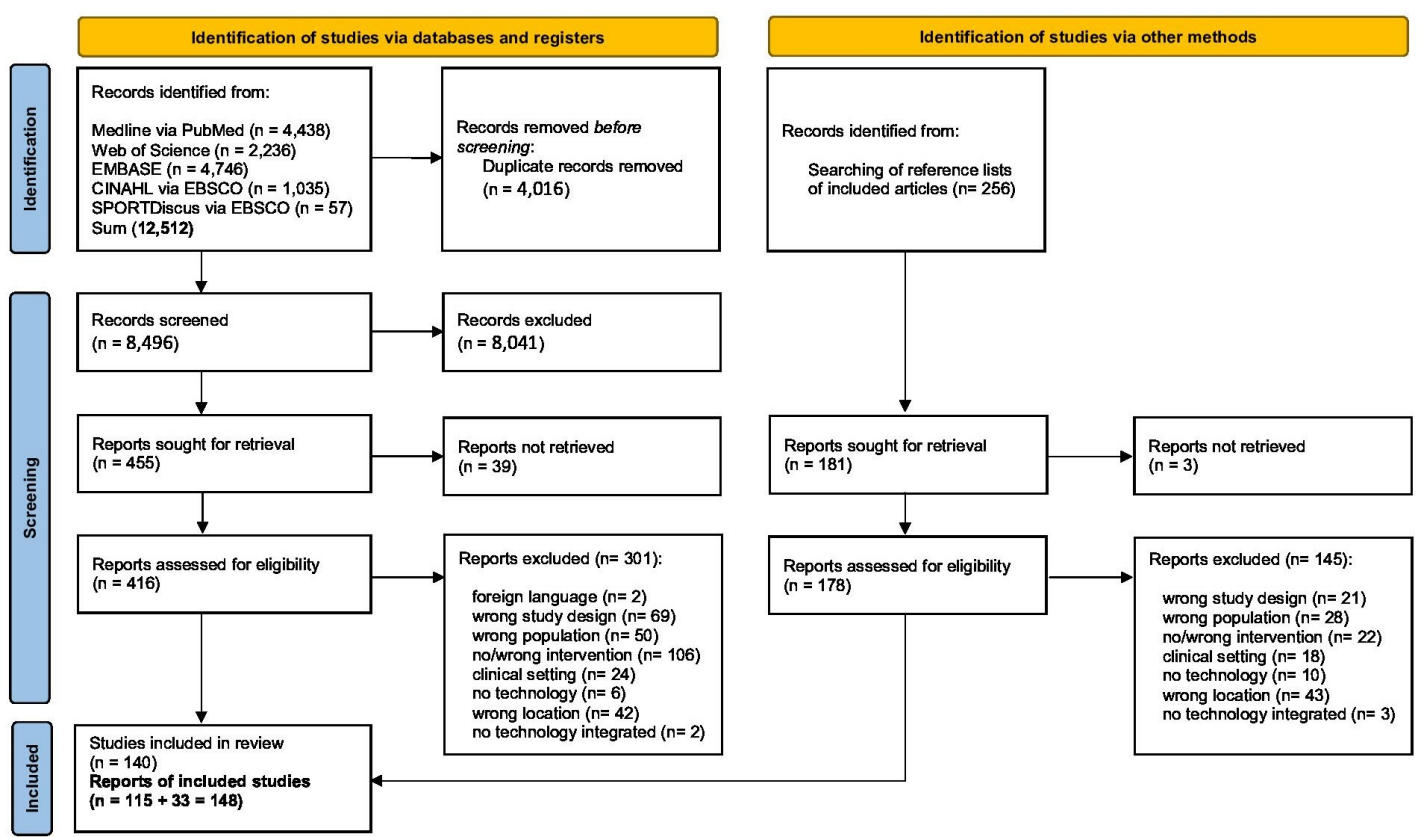
Flowchart search process.

A total of 12,512 sources were identified during searching in Medline (PubMed), Embase (Ovid), CINAHL (EBSCO), SportDiscus (EBSCO), and Web of Science databases. After screening, 148 sources remained. Inclusion and exclusion details can be found in the PRISMA flowchart (Figure 1). An overview of all excluded sources with reasons and all included sources and corresponding extracted data can be found in the supplementary material. Detailed results from the data analysis and the references to the 148 included sources can be found in Multimedia Appendix 4 [33-175]. The full data extraction table and reasons for source exclusions from this review can be found in MultimediaAppendices 5  6, respectively.

### Study Characteristics

**Figure 2.**
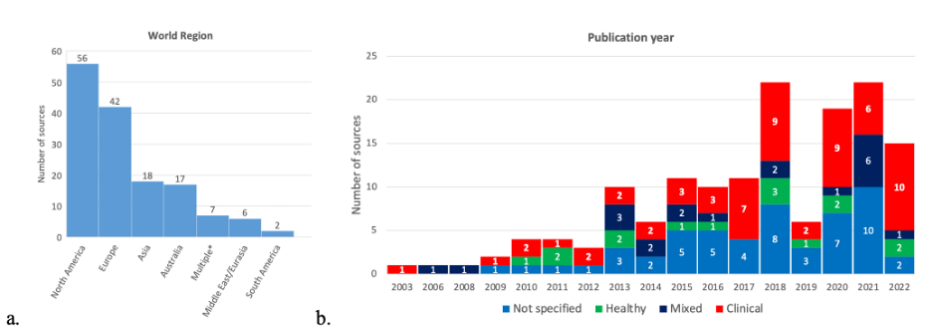
(**A**) Number of included sources by world region. (**B**) and by publication year and health status.

Publication years ranged from 2003 to 2022, with most of the included sources published in the last five years (84/148, 57%). An overview of the number of sources performed by region and publication year is presented in Figure 2.

The sources included 51% (75/148) randomized controlled trials, 24% (35/148) pre-post-test, and 12% (17/148) feasibility studies and to a lesser extent qualitative studies (6/148, 4%), non-randomized controlled trials (5/148, 3%), experimental studies (5/148, 3%), mixed methods (4/148, 3%), and one case study (1/148, 1%).

### Participant Characteristics

The included sources contained a total sample of 12,717 participants, aged 74 (SD 6) years on average, with a range of 60‐88 years. Most sources were devoted to the youngest-old group of 60 to 74 years (87/148, 59%), followed by the middle-old group of 75 to 84 years (51/148, 35%) and the oldest-old group of 85 years and older (6/148, 4%). A small sample of sources reported on mixed (2/148, 1%) age categories. In 2 studies, the age of the participants was not reported. With increasing age groups, the percentage of women increased from 53% (5160/9802) (youngest-old) to 70% (1750/2510) (middle-old) and 79% (119/150) (old-old).

**Figure 3.**
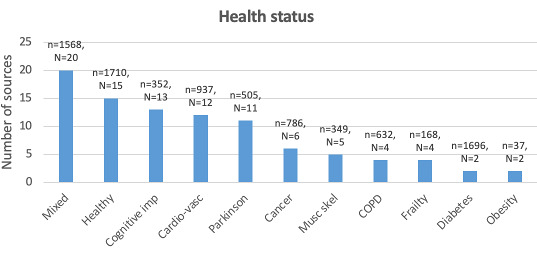
Number of included sources (N) by reported health status. With n being the total number of participants with cardiovascular disease (Cardio-vasc); cognitive impairments (Cognitive imp); chronic obstructive pulmonary disease (COPD) and musculoskeletal disease (Musc skel).

A larger focus on the clinical population is visible since 2017, although the health status is not always properly reported (Figure 2B). In total, 63% (93/148) of the sources reported on the population’s health status (Figure 3). In 37% (55/148) of the sources, information on comorbid conditions was indicated, the average number of comorbidities was 4.2 (SD 2.6; range 1‐10).

Most sources (101/148, 68%) included participants living in community-dwelling residences, 26% of the sources included participants from care homes, while 13% (19/148) of the sources were not specific on participant living arrangements (Figure 3). No source reported an intervention in a hospital setting.

Prior experience with technology was stated in 16% (24/148) of sources, and 15% (22/148) of sources stated that participants had access to technology at home before the implementation of the intervention. Five percent of the sources (7/148) highlight information about the prior frequency of use of these services or devices.

### Intervention Design Characteristics

In 44% (65/148) of the sources, participants received supervised interventions (professional or non-professional) with a further 55% (82/148) of the sources including unsupervised interventions. Additionally, one source tested supervised versus unsupervised technology-based interventions.

In most of the sources (93/148, 63%), the intervention was tailored to individuals and/or groups of participants. The other 37% (55/148) applied a generic intervention approach to all participants. In the tailored interventions, the physical activity was adapted to the participant’s specific needs, or the progression of the activity was personalized by adapting the difficulty, intensity, or frequency level of the activity. Furthermore, individual assessments and feedback systems allowed for continuous adjustment of activities based on performance.

**Figure 4.**
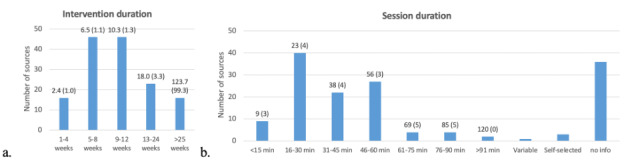
Number of sources stratified by (A). intervention duration (average and SD) and (B) session duration (average and SD). For intervention duration in minutes (min), the average duration and corresponding standard deviation per group is also presented.

Intervention durations varied from one week up to 288 weeks (Figure 4A). A total of 2 interventions (2/148, 1%) did not report intervention duration. Session duration varied by study from 5 minutes up to 120 minutes (Figure 4B). Session frequency was reported in 119 studies and ranged between one to 21 per week. In 2 studies, the weekly session frequency could be chosen independently. Most sources (87/148, 59%) ranged from one to three sessions per week.

The activity intensity was described in only 14% (20/148) of the sources. Of these, 3% (5/148) used Rate of Perceived Exertion, 2% (3/148) the Borg scale, with a further 6% (9/148) of the sources reported intensity in a different manner, eg, the level of the game. Only one source (1/148, 1%) set the intensity at 60% of Heart Rate Reserve, with another 2 sources (2/148,1%) using self-pacing of intensity.

In total, 2 sources (2/148, 1%) did not report on the physical activity used as part of the intervention. The other 146 sources (146/148, 97%) reported 2 to 5 different purposes for the interventions resulting in a total of 296 different purposes. In these interventions, the top 5 purposes were balance (73/148, 49%), muscular strength and power (64/148, 43%), cardiorespiratory fitness (30/148, 20%), functional mobility (28/148, 19%) and general physical activity (25/148, 17%). In 40% (58/148) of the sources, the focus was on one purpose, whereas the majority (88/148, 59%) had on average 3 (min 2, max 5) purposes.

### Technology Characteristics

An overview of the technology designs, functions, and interfaces used in the sources is given in Table 2. Below results of the cross-tabulation between technology and population or intervention characteristics are described. All detailed results can be found in Multimedia Appendix 4.

**Table 2.** Extracted counts for the Design, Function, and Interface categories. N equals the total reported for each category, and in brackets, the percentage of the total reported per Design, Function, and Interface, respectively.

Variable	Categories	N (%)
Design		
	Computer-based	20 (9)
	Sound and video recording systems	8 (4)
	Mobile applications (web-based, tablet, mobile phone)	48 (22)
	Web video/ phone call	30 (13)
	Exergames (incl. screen)	68 (30)
	Trackers	21 (9)
	Virtual reality	6 (3)
	Augmented reality	1 (0)
	Wearable devices	14 (6)
	Other	7 (3)
Function		
	Assessment of outcomes	21 (6)
	Providing exercise program information once	28 (8)
	Providing exercise information continuously	102 (27)
	Feedback of exercise	102 (27)
	Real-time health metrics	6 (2)
	Real-time movement data	98 (26)
	Real-time functionality ability	9 (2)
	Other	5 (1)
Interface		
	Graphical interface	94 (27)
	Command line with feedback	24 (7)
	Command line without feedback	13 (4)
	Menu-driven	40 (11)
	(Haptic) touch	6 (2)
	Auditory or visual	61 (17)
	Body movement	93 (27)
	Form-based	1 (0)
	Natural language processing	0 (0)
	Remote driven	14 (4)
	Other	3 (1)

### Cross-Tabulation Between Technology and Population

The results of the cross-tabulation between participant characteristics and technology design are presented in Table 3. The cross-tabulation results between participant characteristics and technology function and interface can be found in Multimedia Appendix 4. Exergames were implemented in half of the sources with oldest-old adult participants who are aged 85 years and older. In the sources with middle-old age groups (75‐85 y), exergames (25/75, 33%) and mobile apps (16/75, 21%) were both frequently used, and alternative designs such as web/video phone call (10/75, 13%) were implemented as well. In sources with youngest-old age groups (60‐74 y), trackers (18/125, 14%) were more often implemented than in the middle-old or oldest-old age groups (2/75, 3% and 1/18, 6% respectively). In sources where the participant’s health status was reported as healthy, 53% (10/19) implemented exergames compared to only 27% (34/127) in sources with participants with a clinical condition. For both health status groups, mobile apps (20%, ie., 4/19 and 24/127) and trackers (11%, ie., 2/19 and 14/127) were likewise implemented. However, sources with participants with a clinical condition also made use of other technological designs such as computer-based (14/127, 11%), web video/phone call (19/127, 15%) or wearable devices (10/127, 8%). Age and health status did not influence the technological functions or user interface.

**Table 3.** Cross-tabulation results for participant characteristic age, health status, and residency versus technology design.

Design	Sum^a^	Computer based	Sound/ video recording systems	Mobile apps	Web/ video phone call	Exer- games	Trackers	Virtual reality	Augmented reality	Wearable devices	Other
AGE											
Youngest old (60‐74 y)	125	7%	2%	24%	15%	25%	14%	2%	-	6%	4%
Middle old (75‐85 y)	75	11%	7%	21%	13%	33%	3%	4%	-	7%	1%
Oldest old (>85 + y)	18	11%	6%	11%	6%	50%	6%	-	-	6%	6%
Mixed	3	-	-	-	-	67%	-	-	33%	-	-
HEALTH STATUS											
Healthy	19	-	-	21%	5%	53%	11%	5%	-	-	5%
Clinical condition	127	11%	5%	19%	15%	27%	11%	2%	-	8%	3%
RESIDENCY											
Care Homes	42	10%	5%	7%	2%	50%	7%	10%	-	2%	7%
Community Dwelling	153	9%	3%	24%	18%	26%	10%	1%	1%	5%	3%
Hospital	1	-	-	-	-	-	100%	-	-	-	-
Other	33	6%	3%	24%	6%	21%	3%	18%	-	18%	-

a The horizontal rows add up to 100% of the sum in the second column. The other columns represent the percentages of the sum, and the total of each row equals 100%. With abbreviations: Apps, applications; cmnd, command; record., recording; y, years.

In community setting studies, exergames (40/154, 26%) and apps (37/154, 24%) were equally deployed. However, in care homes, half of the sources used exergames. Wearable technology was used frequently in settings where residency was unfortunately not specified, whereas the use of web video/phone call designs was highest (relatively) in community setting studies. Making use of specific functions and interfaces was similarly distributed for community dwelling and care home residents. Only the body movement interface was 34% (21/61) in care homes compared to 25% (59/236) in community settings, probably related to the implemented exergames.

### Cross-Tabulation Between Technology and Intervention

The results of the cross-tabulation analyses for purpose of the physical activity and technology are presented in Multimedia Appendix 4. Exergame and mobile app designs were used to achieve improvements for a large range of physiological parameters such as balance, muscle strength, functional mobility, or cardiovascular health. Similar for web video/phone calls, but these technologies were incorporated into the sources to a much lesser amount. Exergames were predominantly used to improve cardiorespiratory fitness (17/41, 41%), neuromotor function (14/29, 48%), mental and cognitive function (16/32, 50%), and fall risk (8/14, 57%). Only enhancement of general physical activity was trained most using trackers (13/39, 33%, eg, for step counting). Regardless of the purpose, the technologies provided continuous information on the activity, feedback, and assessed real-time movement data. These technological functions were achieved through graphical (25%‐30%), body movement (20%‐36%), or auditory and visual (13%‐23%) interfaces with the users.

Tailoring of the intervention’s physical activity was observed across all technologies. Hence, incorporating tailoring of the intervention’s physical activity did not seem to be related to the type of technology design, function of user interface, and vice-versa.

### Outcome Measurements

#### Adverse Events

In total, 64% (68/148) of the sources reported information on adverse events. Out of these, 72% (49/68) of the sources reported no adverse events, with the other 28% (19/68) reporting a total of 78 adverse events (n=78, 100%) categorized in grade 1 (mild, 40/78, 51%), grade 2 (moderate, 10/78, 13%), grade 3 (severe, 28/78, 36%) events, with none reported in grade 4 (life-threatening) or grade 5 (death).

Of these 78 adverse events, 47 events could be related to the intervention and included 37 mild events, e.g., primarily musculoskeletal pain in joints, 8 moderate events, e.g., rib fracture or COPD-related, and 2 severe events, eg, severe injury due to (bicycle) fall accident. Only 2 adverse events were related to technology and included mild impairments from a skin rash due to Fitbit wearing and nausea due to the Virtual Reality environment.

#### Dropout and Adherence

Information on dropout and adherence for the intervention groups was reported in 100 sources (68%) and 79 sources (53%), respectively. Information on dropout and adherence for the control groups was reported more sparely in 45 sources (47%) and 16 sources (17%), respectively. While the number of participants for all sources is variable, dropout rates of control groups (14 SD 12%) and intervention groups (16 SD 16%) were similar (t_34_ = −0.648, *P*=.521). Average adherence tended to be lower in the control groups (71 SD 25%) compared to the intervention groups (80 SD 18%), though this could not be tested statistically due to underreporting in the control group.

In total, the dropout reasons for 815 participants were quantitatively reported. Reasons for dropout were mostly health-related (28%), though these were unrelated to the intervention. Furthermore, dropout participants mentioned time-related issues (17%), such as lack of time or traveling and burden or motivation-related issues (15%) such as lost interest or feeling burdened. Often another reason (17%) was indicated for the dropout, or no reason was mentioned at all (15%). About 5% of the dropout participants were lost due to follow-up issues ascribed to e.g., loss of contact or moving. Only 3% of the dropouts were related to technology: for example, broken computer, internet malfunctioning, lack of device or space.

No differences (t_98_=−1.209, *P*=.115, *d*=−0.252) were found in dropout when interventions were tailored (15 SD 16%) or not tailored (19 SD 14%). A significantly higher adherence (t_77_=1.995, *P*=0.025, *d*=0.455) was found between interventions that were tailored (83 SD 15%) versus those that were not (75 SD 21%). On the contrary, no differences were observed when comparing supervised versus unsupervised interventions, when assessing dropout (t_98_=-1.374, *P*=.086, *d*=−0.275) and adherence (t_77_=0.606, *P*=.273, *d*=0.136).

Sources including participants with reported clinical conditions as health status (18 SD 11% dropout) reported a similar level of dropout rate compared to those who used healthy populations (17 SD 18% dropout), which was nonsignificant (t_62_=0.063, *P*=.475, *d*=0.022). There were no differences between adherence rates when comparing the sources with reported healthy participants versus participants with a reported clinical condition (t_48_=0.027, *P*=.489, *d*=0.010). Differential testing between age groups was not possible, due to the low reported dropout ratios across three age categories, although no trends were observed of dropout rate differences across age categories.

No further relationships were found between dropout or adherence, when regressing against the total number of intervention sessions (*R*^2^=0.012, *P*=.924) or session duration (*R*^2^=0.001, *P*=.850).

#### User Experience

A total of 55 sources (37%) explored the general enjoyment of the technology part of their intervention. An overwhelming 91% reported a positive experience, with a vast minority reporting a neutral or negative experience (7% and 2% respectively).

Only 21 sources (14%) investigated the system usability. The system usability was reported using the System Usability Scale (SUS, N=12, 8%) or other questionnaires, mostly using Likert scales or percentages (N=9, 6%). The reported SUS values ranged from 62 to 90 with an average of 77 (SD 9) indicating okay, good, and excellent overall user friendliness [176]. In several sources (N=2), it was reported that the SUS was assessed, but then the result was not presented.

Regardless of the study design, wishes and needs of the users were seldom (systematically) assessed. When reported, the wishes were related to goal setting, feedback (regarding own or other participants' performance, messages), support (technical, medical), the physical activity (variation, choice of level), the technology (access, comfort, malfunctioning, touch screen), and social setting (in groups, partnering up, avatar look, timing).

## Discussion

### Principal Findings

This scoping review explores the application of digital technologies in relation to the characteristics of populations (eg, health status), interventions and usability in home-based physical activity interventions for older adults. Analyzing 148 sources, the review finds that technologies like exergames and mobile apps are widely applicable and easy to use across diverse older adult populations, including healthy and those with clinical conditions. These technologies offer continuous features like information about the physical activity programs, feedback, and real-time movement tracking, addressing various goals such as balance, strength, functional mobility, and cardiovascular fitness. Interestingly, technology use did not lead to higher dropout rates, and adherence was significantly higher in tailored interventions. Adverse events directly related to the technology were rare. User evaluations indicated positive feedback regarding the usability and enjoyment of the technology, suggesting its potential to increase physical activity engagement among older adults.

### Participants

Our research underscores the growing evidence of technology-based physical activity interventions in home-based environments (community dwelling and care homes) since 2010, with a notable increase over the past five years [177]. Our review revealed that technology-assisted physical activity interventions have been explored among older adults in various age groups and with various health conditions. Given the increasing prevalence of age-related diseases and chronic health conditions among older adults, technology holds promise in promoting physical activity engagement, particularly among those with health challenges [19,25].

The use of technology-assisted physical activity interventions for older adults in hospital settings is limited [178-180]. Initially, this review identified no such studies (N=0), likely due to excluding abstracts mentioning “rehabilitation” in our search. While physical activity interventions in hospitals generally show positive effects on functional decline or hospital-associated disability [3,181,182], Sheerman et al report inconsistent findings [183]. Similarly, technology-assisted interventions in hospitals report mixed results: adherence was high for supervised interactive gaming [180] and activity trackers [178] but low for self-regulated exergames [179]. Furthermore, Laver et al reported improved Time-Up-and-Go test results with no serious adverse events, while Oesch et al noted no balance improvements and 2 adverse events related to pain [179,180].

Studies failed to report participants’ education (only 40 %) and socio-economic status (only 9 %), which are critical factors as lower education and lower socioeconomic status often correlate with lower digital health literacy [184,185]. Lower education influences the level of health literacy in general as well [184], which may influence judgment on the relevance of engaging with health technology [186]. Furthermore, lower socio-economic status has been related to suboptimal access to internet and digital tools [184], e.g., due to costs [187]. Unfortunately, only limited information was reported by the included sources (15%) on access to technology. Also, technology familiarity or e-literacy was poorly reported (15%). Lower education and the lack of familiarization with technology may introduce barriers to the capacity of users to understand and attend a technology-supported intervention and may lead to increased anxiety and dropouts when attending [186-188]. Hence, the limited reporting on such participant characteristics raises concerns about sample bias and the generalizability of results.

### Interventions

This scoping review reveals significant diversity in technology-assisted intervention designs, supervision levels, and tailoring. While many interventions are appropriately tailored to the specific needs of older adults and target populations, this tailoring naturally results in variability in intervention durations, session lengths, and frequencies. This does not necessarily indicate a lack of structure but reflects the necessary flexibility in designing interventions that meet individual or group needs. However, the lack of consistent reporting frameworks for these interventions poses a challenge for comparing results across studies. Our findings are consistent with other reviews in the field (eg, [177,189-191]), which also identified significant variability in intervention designs and reporting frameworks. Those reviews, like ours, emphasize the importance of individualization and tailoring as the key components in ensuring that interventions are effective, but the absence of standardized reporting makes it difficult to evaluate the relative success of these programs. Particularly, the underreporting of intervention intensity poses a significant challenge, with only 14% of sources detailing intervention intensity using varied methods. This is unexpected since technology can easily track and report intensity, providing both motivation and precise data for older adults. For instance, studies using Kinect systems and wearables like Fitbit have shown potential in accurately tracking activity intensity, enhancing engagement and usability [192-194].

### Technologies

In the sources we reviewed, exergaming emerged as the most used approach, featuring 53% of interventions for healthy populations and 27% for clinical groups. Despite most exergaming applications targeting younger populations [195,196], our scoping review identifies a significant shift towards older users, with exergames implemented in half of the sources involving participants aged 85 and above. The findings support and build upon previous research suggesting the utility of exergames in promoting physical activity, cognitive enhancement, and overall well-being in the older population [11]. Furthermore, the diversity of exergame intervention contents highlights a comprehensive strategy for addressing age-related challenges such as cardiorespiratory fitness (41%), neuromotor functions (48%), mental and cognitive abilities (50%), and reducing falls (57%).

Our findings also indicate that while exergames and mobile applications are the primary technologies used in these physical activity interventions, web video and phone call technologies or wearable devices also play a role, albeit less prominently. Especially in the clinical population, more often other designs than exergames were used compared to the interventions performed with healthy older adults. These other forms of technology design, offering a more passive interaction than the dynamic engagement found in exergames, may not be as prevalent but are nonetheless vital. Older, or more prone older adults may prefer and benefit from their easy set-up and simple usage [197-199]. Their integration highlights their significance within a comprehensive technological intervention framework, which aligns with effective telehealth components like text messaging for education and reminders, web-based content for goal-setting, and interactive platforms for health data exchange [200].

In advocating for the integration of technology in physical activity interventions, our review highlights the pivotal role of technology functions and user interfaces, as supported by Bentlage et al. (2023), who stress their essential contribution to improve the activation factors skills, knowledge, and motivation, yielding effective behavioral change [20]. We pointed out the primary technological functions as the continuous provision of physical activity information (28%), performance feedback (28%), and the recording of real-time movement data (26%). Such continuous and synchronous activity information offers users an immersive, enjoyable, and engaging experience [201], fosters regular habits [202], can facilitate the visualization of their movements, and significantly increase their motivation [203]. Enhancing user engagement and overcoming engagement barriers can be achieved by using advanced technology to incorporate different user interfaces, preferably multiple types [20,204]. While graphical or body movement interfaces were prevalent in many sources (each 63%), alternative user interfaces were limited (ie, 41% visual/auditory, 27% menu driven, and 16% command line).

Notably, the tailoring of intervention exercises or activities did not significantly influence the choice of technology design, function, or user interface. This might indicate that the deployed technological solutions are versatile and adaptable across various intervention needs without requiring significant modifications [205,206]. Moreover, our review emphasizes a shift towards personalized interventions tailored to individual or group needs, with 63% of interventions adopting this approach (versus 37% with a generic approach). This shift aligns with emerging evidence suggesting the effectiveness of personalized strategies in promoting adherence and achieving positive health outcomes among older adults [207].

### Technology-Related Outcomes

Technology engagement and adherence to protocols are tightly linked to the usability, enjoyment, and effectiveness of physical activity-based interventions [208]. Yet only a slight majority of studies reported on dropout and adherence rates, whereas an even smaller number reported on reasons for dropout or (reduced) adherence. Despite the Consolidated Standard of Reporting Trials (CONSORT) [209] statement and TIDieR (Template for Intervention Description and Replication) [32] guidelines dating back to 2010 and 2014, and the relevant Standard Protocol Items: Recommendations for Interventional Trials (SPIRIT) 2022 extension [210], there is still a lack of consistency of reporting.

Sources that reported the reasons for dropout among participants primarily cited health-related issues, with only a few instances attributed to technology usage, underscoring the older participants’ willingness and engagement with home-based technology. Such instances of technology-related dropout (3%) were predominantly due to technological failures, connectivity issues, or general disinterest. These findings are consistent with recent studies, which also identified a lack of motivation and low familiarity with technology as key barriers to its implementation in older adults [15,23,211]. Furthermore, tailored interventions resulted in significantly higher adherence than control interventions. This underscores that it is imperative to adapt the technology to the preferences and needs of the target aging population [22,23,197-199,212-214]. To achieve suitable technological approaches, a generative co-design framework, could be used to overcome technological barriers in an aging population, as suggested by Bird et al [215].

### Strengths and Limitations of This Scoping Review

Adopting a common vocabulary and definition of terms is challenging in research on technology-assisted physical activity interventions, which can be the intersection of medicine, physiotherapy, engineering and informatics, sensor and gaming technology, and even psychology and social sciences. Though challenging, we discussed and agreed upon definitions and vocabulary usage at each phase of the review. Furthermore, multiple protocols and double-blind scoring were incorporated in most phases of the review process to ensure consistency [216]. The relatively large number of screeners and extractors involved may be considered a potential limitation. However, the screening, data extraction, and data analysis tools were carefully prepared and discussed within the group, and conflicts were solved with a third independent reviewer or group discussions.

Regarding the included sources, most of the reviewed studies were from highly developed and advanced countries. In addition, the sources included in this scoping review often failed to describe the used technology in sufficient detail to allow for replication of the design, function, and/or user interface. For example, exergames rely on an often bilateral interaction between the user and the system. However, the inputs (eg, handheld devices, sensors, video cameras) are poorly described on how these were adapted for use. Valenzuela et al 217 explored older adults’ experiences using an interactive cognitive-motor step training program. Yet, there is no clear description of how the interface has been adapted for their specific user group. Other sources assumed prior or even in-depth knowledge of commercial exergaming setups used within the intervention, avoiding interpretation of the data or replicability due to unknown use of commercial games (Wii-Fit), body movement recognition through three-dimensional camera setups (Xbox Kinect), or not clearly described engagement elements of online video calling for physical activity class delivery (eg, Yoga via Zoom calls) [218,219].

This review has been carried out in a systematic and transparent manner [220]. However, we have not conducted a quality assessment or risk of bias of the included sources. Therefore, we cannot use the results of the included sources to draw significant conclusions on intervention effects on health outcomes.

### Recommendations

There is no compelling reason to exclude specific older populations from home-based, technology-assisted physical activity interventions, as factors such as age, health status, and residency do not appear to negatively impact dropout rates, adherence, or result in technology-related adverse events. However, evidence regarding the use of such technologies in hospital settings remains limited. Vulnerable older adults, in particular, could benefit significantly from these interventions, as reduced physical activity during hospitalization often leads to functional decline and a loss of independence in daily activities at discharge. Regardless of the setting, the design of technology-assisted interventions must account for the physical and cognitive impairments commonly observed among older adults, which may result in a mismatch between their abilities and the demands of the intervention or technology [186]. Careful tailoring of both the intervention and the technology is therefore essential. For example, participatory design processes that engage older adults directly can ensure better alignment with their needs and preferences [215]. Our review highlights that tailored approaches are associated with significantly higher adherence rates. Furthermore, feedback from older adults highlights their strong preference for tailored approaches, indicating that tailoring is not only beneficial but also actively desired by older adults. To improve the accessibility and impact of these interventions, training to enhance health literacy (knowledge of the intervention’s relevance) and digital literacy (competence in using the technology) should be incorporated during both the recruitment and intervention phases. Developing clear recruitment guidelines may further promote inclusivity. Digital literacy barriers, often linked to low education levels and socioeconomic status, remain significant challenges to technology adoption and engagement [184,186-188]. To address this, technical support—either private or as part of the intervention—should be provided, aligning with preferences expressed by older adults in user feedback. Lastly, behavioral change techniques that enhance skills, knowledge, and motivation should be integrated into the design of technology-assisted physical activity interventions [20]. Feedback mechanisms, such as performance-based metrics, functional ability assessments, and real-time health data (eg, movement intensity), are currently underutilized, appearing in less than one-third of interventions. Implementing such features could encourage behavioral change and fulfill the desires for actionable feedback, as reported by older adults.

Furthermore, future research should prioritize transparent reporting. The participant’s health status, including co-morbidities, mental and physical impairments, and activity level, as well as technology experience, should be recorded. The intervention design, including purpose, duration, intensity, supervision, and tailoring, should be well described. The technology design, functions (including consideration of behavioral change techniques), and user interface should be described in detail. Lastly, intervention and technology-related outcomes such as drop-out, adherence, adverse events, and feedback on user experience should be reported. Guidelines for the development of technology-assisted physical activity interventions in older adults with various health conditions may lead to conformity and could contribute to better designs engaging older adults. Such guidelines may improve the effectiveness of the interventions and make studies better comparable by adequate reporting. The latter results in a deeper understanding of user preferences and advances the development of tailored technology-assisted interventions.

### Conclusions

The review suggests that using technology in physical activity interventions is feasible for all older adults in community dwellings and care homes, without additional risks of adverse events or dropouts. Furthermore, generally higher adherence was reported in technology-assisted interventions, which was significantly higher where interventions were tailored. Interventions were delivered to all older age groups, as well as those with and without clinical conditions and in a wide range of physical activity topics. The technology approaches for home-based physical activity interventions were found to be safe and enjoyable for older adults with acceptable usability. Despite the large number of sources that have been identified in this article, the lack of standardized reporting limited a more in-depth analysis of the main research question. Therefore, there is a need to update reporting guidelines for technology-specific interventions in this area.

## Supplementary material

10.2196/65746 Multimedia Appendix 1 Search string development.

10.2196/65746 Multimedia Appendix 2 Data extraction topics.

10.2196/65746 Multimedia Appendix 3 Coding framework for data extraction.

10.2196/65746 Multimedia Appendix 4 Result tables and sources.

10.2196/65746 Multimedia Appendix 5 Full data extraction.

10.2196/65746 Multimedia Appendix 6 Excluded sources reasons.

10.2196/65746 Checklist 1 PRISMA-ScR checklist.

## Abbreviations

CONSORT: Consolidated Standard of Reporting Trials
COST: Cooperation in Science and Technology
JBI: Joanna Briggs Institute
PRISMA-ScR: Preferred Reporting Items for Systematic Reviews and Meta-analyses extension for scoping reviews
SPIRIT: Standard Protocol Items: Recommendations for Interventional Trials
