# Supplementary material for: Technology-Assisted Physical Activity Interventions for Older People in Their Home-Based Environment: Scoping Review
Source: JMIR Aging. 2025 Sep 15;8:e65746. doi: 10.2196/65746 (PMC12516299; doi:10.2196/65746)
Supplement: Multimedia Appendix 1 [file aging-v8-e65746-s001.docx]

**APPENDIX 1: SEARCH STRING DEVELOPMENT**

1. **Identification of keywords**

(Based on Medical Subject Headings 2022 Browser - <https://meshb.nlm.nih.gov/>)

| **No** | **MeSH terms** | **Area covered in MeSH terms** | **Non-MeSH terms** |
| --- | --- | --- | --- |
| 1 | adult  AND | **Adult ->aged and** [**aged, 80 and over**](https://meshb.nlm.nih.gov/record/ui?ui=D000369):  elderly (elderly, frail; frail elders;  **Adult ->aged and** [**aged, 80 and over**](https://meshb.nlm.nih.gov/record/ui?ui=D000369):  elderly (elderly, frail; frail elders;  frail older adults, functionally-impaired elderly); older | aged;  elder*;  aged;  elder*;  old;  older people;  geriatric;  senior# |
| 2 | home environment  OR | **home environment**: living alone;  social housing conditions | home*  resident*  care facilit*  nursing home* |
| 3 | independent living  OR | **independent living**: aging in place; community dwelling |  |
| 4 | residential facilities  AND | **residence facilities->**long-term care facilities; assisted living facilities; group homes; halfway houses;  homes for the aged |  |
| 5 | technology  OR | **technology**: digital technology; biomedical technology -> health technology | digital technolog*;  mobile health;  telehealth;  eHealth;  mHealth;  exergam*;  video gam*;  serious gam*;  wearable technolog*;  wearable electronic device*;  application*;  mobile app*;  virtual reality |
| 6 | telemedicine  OR | **telemedicine**: mobile health; telehealth; eHealth; mHealth |  |
| 7 | wearable electronic devices  OR | **wearable electronic devices**: wearable devices; wearable technology;  wearable diagnostic devices; wearable [activity trackers](https://meshb.nlm.nih.gov/record/ui?ui=D000072936); fitness trackers |  |
| 8 | mobile applications  OR | **mobile applications**: mobile apps;  portable electronic applications; portable electronic apps; portable software applications; portable software apps; smartphoneapps |  |
| 9 | virtual reality  AND | **virtual reality**: virtual reality, educational; virtual reality, instructional |  |
| 10 | Humanactivities  OR | **human activities**:  Exercise ->exergaming;  leisure activities -> recreation-> dancing, sports, play and playthings | exercise#;  training;  intervention*;  physical activit*;  physical fitness |
| 11 | exercise  OR | **exercise**: acute exercise; aerobic exercise; exercise training; isometric exercise; physical activity;  -> exergaming |  |
| 12 | physical fitness | **physicalfitness**:cardiorespiratory fitness |  |

**Exclusion -** NOT (review[Title] OR rehabilitation[Abstract])

**2. Keywords strings established to adopt for search in specific databases**

a) MeSH terms – “locked” only for search in the MeSH tree

((adult[MeSH Terms]) AND ((home environment[MeSH Terms]) OR (independent living[MeSH Terms]) OR (residential facilities[MeSH Terms])) AND ((technology[MeSH Terms]) OR (telemedicine[MeSH Terms]) OR (wearable electronic devices[MeSH Terms]) OR (mobile applications[MeSH Terms]) OR (virtual reality[MeSH Terms])) AND ((human activities[MeSH Terms]) OR(exercise[MeSH Terms]) OR (physical fitness[MeSH Terms]))NOT (review[Title/Abstract] OR rehabilitation[Title/Abstract]))

b) MeSH terms – “unlocked” for search in the MeSH tree and All Fields

adult AND (home environment OR independent living OR residential facilities) AND (technology OR telemedicine OR wearable electronic devices OR mobile applications OR virtual reality) AND (human activities OR exercise OR physical fitness) NOT ("review"[Title/Abstract] OR "rehabilitation"[Title/Abstract])

c) non-MeSH terms

(“aged” OR “elder*” OR “old” OR “older people” OR “geriatric” OR “senior#”) AND (“home*” OR “resident*” OR “care facilit*” OR “nursing home*”) AND (“digital technolog*” OR “mobile health” OR “telehealth” OR “eHealth” OR “mHealth” OR “exergam*” OR “video gam*” OR “serious gam*” OR “wearable technolog*” OR “wearable electronic device*” OR “application*” OR “mobile app*” OR “virtual reality”) AND (“exercise#” OR “training” OR “intervention*” OR “physical activit*” OR “physical fitness”) NOT (“review”[Title/Abstract] OR “rehabilitation” [Title/Abstract])

**3. Keywords strings used in specific databases (with needed adaptations)**

**PubMed**

1. Unlocked MeSH – search in MeSH tree and All Fields

adult AND (home environment OR independent living OR residential facilities) AND (technology OR telemedicine OR wearable electronic devices OR mobile applications OR virtual reality) AND (human activities OR exercise OR physical fitness) NOT ("review"[Title/Abstract] OR "rehabilitation"[Title/Abstract])

1. Non-MeSH – search in All Fields

(“aged” OR “elder*” OR “old” OR “older people” OR “geriatric” OR “senior#”) AND (“home*” OR “resident*” OR “care facilit*” OR “nursing home*”)  AND (“digital technolog*” OR “mobile health” OR “telehealth” OR “eHealth” OR “mHealth” OR “exergam*” OR “video gam*” OR “serious gam*” OR “wearable technolog*” OR “wearable electronic device*” OR “application*” OR “mobile app*” OR “virtual reality”) AND (“exercise#” OR “training” OR “intervention*” OR “physical activit*” OR “physical fitness”) NOT (“review”[Title/Abstract] OR “rehabilitation” [Title/Abstract])

**Web of Science**

Non MeSH – search in All Fields

(“aged” OR “elder*” OR “old” OR “older people” OR “geriatric” OR “senior#”) AND (“home*” OR “resident*” OR “care facilit*” OR “nursing home*”)  AND (“digital technolog*” OR “mobile health” OR “telehealth” OR “eHealth” OR “mHealth” OR “exergam*” OR “video gam*” OR “serious gam*” OR “wearable technolog*” OR “wearable electronic device*” OR “application*” OR “mobile app*” OR “virtual reality”) AND (“exercise#” OR “training” OR “intervention*” OR “physical activit*” OR “physical fitness”)

+ NOT “review” in [Title]

+ NOT “rehabilitation” in [Abstract]

**EMBASE**

1. Non-MeSH – search in All Fields

('aged' OR 'elder*' OR 'old' OR 'older people' OR 'geriatric' OR 'senior*') AND ('home*' OR 'resident*' OR 'care facilit*' OR 'nursing home*') AND ('digital technolog*' OR 'mobile health' OR 'telehealth' OR 'ehealth' OR 'mhealth' OR 'exergam*' OR 'video gam*' OR 'serious gam*' OR 'wearable technolog*' OR 'wearable electronic device*' OR 'application*' OR 'mobile app*' OR 'virtual reality') AND ('exercise*' OR 'training' OR 'intervention*' OR 'physical activit*' OR 'physical fitness') NOT review:ti,ab NOT rehabilitation:ti,ab

1. Emtree term “exploded” – search in Emtree (adaptation of MeSH term string)

adult AND (home environment OR independent living OR residential home) AND (technology OR telemedicine OR wearable computer OR mobile applications OR virtual reality) AND (human activities OR exercise OR fitness) NOT review:ti,ab NOT rehabilitation:ti,ab

« residential facilities » is replaced by « residential home »

« wearable electronic device » is replaced by « wearable computer »

« physical fitness » is replaced by « fitness »

**SportDisscus**

Non-MeSH – search in All Fields

(“aged” OR “elder*” OR “old” OR “older people” OR “geriatric” OR “senior#”) AND (“home*” OR “resident*” OR “care facilit*” OR “nursing home*”)  AND (“digital technolog*” OR “mobile health” OR “telehealth” OR “eHealth” OR “mHealth” OR “exergam*” OR “video gam*” OR “serious gam*” OR “wearable technolog*” OR “wearable electronic device*” OR “application*” OR “mobile app*” OR “virtual reality”) AND (“exercise#” OR “training” OR “intervention*” OR “physical activit*” OR “physical fitness”)

+ NOT “review” in [Title]

+ NOT “rehabilitation” in [Abstract]

**CINAHL**

Non-MeSH – search in All Fields

(“aged” OR “elder*” OR “old” OR “older people” OR “geriatric” OR “senior*”) AND (“home*” OR “resident*” OR “care facilit*” OR “nursing home*”) AND (“digital technolog*” OR “mobile health” OR “telehealth” OR “eHealth” OR “mHealth” OR “exergam*” OR “video gam*” OR “serious gam*” OR “wearable technolog*” OR “wearable electronic device*” OR “application*” OR “mobile app*” OR “virtual reality”) AND (“exercise*” OR “training” OR “intervention*” OR “physical activit*” OR “physical fitness”) NOT (TI review OR TI

 review) NOT (TI rehabilitation OR AB rehabilitation)

**4. Example of step by step records retrieval (non-MeSH string, CINAHL, 5.10.2022)**

| **#** | **Search** | **Records retrieved** |
| --- | --- | --- |
| S1 | "aged" | 1,085,824 |
| S2 | “elder*” | 9,914 |
| S3 | “old” | 224,350 |
| S4 | “older people” | 30,679 |
| S5 | “geriatric” | 45,553 |
| S6 | “senior*” | 29,920 |
| S7 | “home*” | 281,748 |
| S8 | “resident*” | 95,302 |
| S9 | “care facilit*” | 31,555 |
| S10 | “nursing home*” | 49,003 |
| S11 | “digital technolog*” | 3,135 |
| S12 | “mobile health” | 22,962 |
| S13 | “telehealth” | 26,498 |
| S14 | “eHealth” | 21,074 |
| S15 | “mHealth” | 20,520 |
| S16 | “exergam*” | 602 |
| S17 | “video gam*” | 6,385 |
| S18 | “serious gam*” | 492 |
| S19 | “wearable technolog*” | 568 |
| S20 | “wearable electronic device*” | 11 |
| S21 | “application*” | 182,272 |
| S22 | “mobile app*” | 12,981 |
| S23 | “virtual reality” | 9,998 |
| S24 | “exercise*” | 210,060 |
| S25 | “training” | 263,828 |
| S26 | “intervention*” | 337,136 |
| S27 | “physical activit*” | 92,702 |
| S28 | “physical fitness” | 21,166 |
| S29 | S1 OR S2 OR S3 OR S4 OR S5 OR S6 | 1,196,497 |
| S30 | S7 OR S8 OR S9 OR S10 | 374,012 |
| S31 | S11 OR S12 OR S13 OR S14 OR S15 OR S16 OR S17 OR S18 OR S19 OR S20 OR S21 OR S22 OR S23 | 227,391 |
| S32 | S24 OR S25 OR S26 OR S27 OR S28 | 769,139 |
| S33 | S29 AND S30 AND S31 AND S32 | 1,350 |
| S34 | TI rehabilitation OR AB rehabilitation | 102,190 |
| S35 | TI review OR AB review | 623,421 |
| S36 | S33 NOT S34 NOT S35 | 1,035 |

**5. Access date and number of records retrieved in searched databases**

| **Database (access date)** | **MeSH/Emtree** | **nonMeSH** | **sum** |
| --- | --- | --- | --- |
| Medline via PubMed (26.09.2022) | 1,757 | 2,681 | 4,438 |
| Web of Science (6.10.2022) | - | 2,236 | 2,236 |
| EMBASE (3.10.2022) | 198 | 4,548 | 4,746 |
| CINAHL via EBSCO (5.10.2022) | - | 1,035 | 1,035 |
| SPORTDiscus via EBSCO (3.10.2022) | - | 57 | 57 |
| **All records** | **1,955** | **10,557** | **12,512** |
