# Supplementary material for: Technology-Assisted Physical Activity Interventions for Older People in Their Home-Based Environment: Scoping Review
Source: JMIR Aging. 2025 Sep 15;8:e65746. doi: 10.2196/65746 (PMC12516299; doi:10.2196/65746)
Supplement: Multimedia Appendix 2 [file aging-v8-e65746-s002.docx]

**APPENDIX 2. DATA EXTRACTION TOPICS**

1. **BIBLIOGRAPHIC**

| **BIBLIOGRAPHIC** |  |
| --- | --- |
| Author(s) |  |
| Reviewer Initials (in case of questions) |  |
| Included or Excluded? |  |
| Year of publication |  |
| Title |  |
| DOI or duplicates avoided using screening software? |  |
| Country of origin (where study is performed) |  |
| Aim/purpose |  |

1. **SUBJECTS**

| **SUBJECTS** |
| --- |
| Gender, age of participants |
| Body Mass Index (BMI), nutritional status |
| Ethnicity and religion |
| Education |
| Economic situation and Job-related activity: **Yes/No**; current/previous sector of work, job, volunteer work |
| Living situation: area (rural/city), residence (independent living = community-dwelling = retirement communities versus Nursing or care-home/hospital), arrangements (living alone, marital status, with (grand)children or care giver), self-care score (e.g. Barthel index). Environment |
| Main clinically diagnosed conditions |
| Co- (or multi-) morbidities (e.g., medication-physical-mental impairments) |
| Mobility and physical activity behaviour |
| Technology use behaviour |

1. **TECHNOLOGY**

| **TECHNOLOGY** | **EXPLANATION** |
| --- | --- |
| Characteristics of technology: design and interface | hardware and software and user interface |
| Characteristics of technology: function and usability | What is role of technology in intervention |
| Characteristics of technology: type of interaction with participants | How does the interface work |

1. **OUTCOME MEASURES**

| **TECHNOLOGY** |
| --- |
| Primary outcome measures |
| Secondary outcome measures |
| User Experience |
| Analysis method/statistics |

1. **KEY FINDINGS**

| **KEY FINDINGS** |
| --- |
| Primary Outcome measure results |
| Secondary outcome measures results |
| Safety (Adverse events happened? Not-foreseen findings?) |
| Dropouts and adherence |
| Factors related with participant interaction: impact on participant (user experience of intervention?) |
| Factors related to the use of technology (user experience of technology?) |
| Barriers: related to health conditions |
