# Supplementary material for: Technology-Assisted Physical Activity Interventions for Older People in Their Home-Based Environment: Scoping Review
Source: JMIR Aging. 2025 Sep 15;8:e65746. doi: 10.2196/65746 (PMC12516299; doi:10.2196/65746)
Supplement: Multimedia Appendix 3 [file aging-v8-e65746-s003.docx]

**APPENDIX 3. CODING FRAMEWORK**

**A. CATEGORIES**

1. **Study design**: randomized controlled trials (RCT), non-randomized controlled trials, pre-and post-test studies, feasibility studies, qualitative studies, experimental studies, mixed methods, case study.
2. Information about **participants**: personal factors (e.g., age, gender, BMI, ethnicity, education, economic situation, and job-related activity), living situation (e.g., area, marital status, residence, self-care ability), health conditions (clinically diagnosed conditions, comorbidities, medication usage), functional ability limitations (mobility, physical activity behaviour).
3. **Intervention** characteristics: intervention details (e.g., provider, setting, activity type, participant recruitment / selection / allocation, main intervention characteristics (e.g., format, time frame, purpose of physical activity, session length and frequency), tailoring/customization/personalization, comparator.
4. **Technology** characteristics: design, interface, function, usability, interaction with participants.
5. **Outcome measures**: primary and secondary technology and health related outcomes, user experience.

**B. DEFINITIONS OF (SUB)CATEGORIES**

**(1) STUDY DESIGN**

**Randomized Controlled Trial**: refers a study where participants are assigned to minimum two separate groups (controlled refers to a presence of a concurrent control or comparator group), each group receives a different intervention (the control group receives no intervention or another intervention, random means that each participant has an equal chance of being allocated to the two groups). It includes inclusion and exclusion criteria [1] .

**Non-Randomized Controlled Trial:** refers to a study where participants are assigned to different intervention arms without following a random procedure [1].

**Pre-post trial:**  refers to a study that measures outcomes (dependent variables) are measured before and after an intervention in the same participants [1].

**Feasibility study:**  refers to a preliminary analysis or investigation conducted to assess the practicality and viability of a proposed project or research question that asks whether something can be done, should we proceed with it, and if so, how [2].

**Qualitative Study:** refers to the study of the nature of phenomena”, including “their quality, different manifestations, the context in which they appear or the perspectives from which they can be perceived”, but excluding “their range, frequency and place in an objectively determined chain of cause and effect” [3].

**Experimental Study**: refers to study where researchers actively perform an intervention in some or all members of a group of participants [4].

**Mixed-Methods Study:** Qualitative methods can be combined with other methods in multi- or mixed methods designs, which employ two or more different methods within the same study or research program. Reasons for combining methods can be diverse, including triangulation for corroboration of findings, complementarity for illustration and clarification of results, expansion to extend the breadth and range of the study, explanation of unexpected results generated with one method with the help of another, or offsetting the weakness of one method with the strength of another [3].

**Case Report**: refers to the description of a patient with an unusual disease or with simultaneous occurrence of more than one condition [5].

**(2) PARTICIPANT CHARCATERISTICS**

**Participant groups.** Refers to intervention and control groups (number of participants, mean age (SD), % females).

**Age groups.** Age has been categorized into 3 groups [6–8]:

- Youngest-old: 60-74 years,
- Middle-old: 75-84 years,
- Oldest-old: ≥ 85 years.

**Body composition.** Refers to Body Mass index (BMI), Height, % Fat.

**Ethnicity.** Categorized according to [9,10]. Standards for Maintaining, Collecting, and Presenting Federal Data on Race and Ethnicity, National Centre of Health Statistics. The following groups defined:

- Asian- A person having origins in any of the original peoples of the Far East, Southeast Asia, or the Indian subcontinent including, for example, Cambodia, China, India, Japan, Korea, Malaysia, Pakistan, the Philippine Islands, Thailand, and Vietnam.
- Hispanic/Latino - A person of Cuban, Mexican, Puerto Rican, South or Central American, or other Spanish culture or origin, regardless of race. The term, "Spanish origin," can be used in addition to "Hispanic or Latino."
- White/Caucasian - A person having origins in any of the original peoples of Europe, the Middle East, or North Africa.
- American Indian/Alaska Native - A person having origins in any of the original peoples of North and South America (including Central America), and who maintains tribal affiliation or community attachment.
- Black/African American - A person having origins in any of the black racial groups of Africa. Terms such as "Haitian" or "Negro" can be used in addition to "Black or African American."
- Native Hawaiian/Pacific Islander - A person having origins in any of the original peoples of Hawaii, Guam, Samoa, or other Pacific Islands.
- Multiracial.
- Mixed populations (with clarification what race is in this population).

**Education level.** Categorized according to National education systems and International Standard Classification of Education [11–13] :

- Low: 0-8 years,
- Middle: 9-11 years,
- High: ≥12 years.

**Economic status**. Employment status and level of income.

Employment:

- unemployed,
- working,
- retired.

And level of income:

- low,
- middle,
- high,
- or mixed.

**Living status**: Describes a person’s residency and personal support:

- independent (community dwelling),
- retirement home,
- nursing-care-hospital,
- with a partner (yes/no),
- or with a caregiver (yes/no).

**Health status**: The reported health status was categorized according to International Classification of Disease and Related Health Problems (ICD-11) [14].

- healthy,
- neoplasms (cancer),
- cardio-vascular disease,
- mental and cognitive disorders,
- respiratory disease (COPD),
- musculoskeletal system disease,
- metabolic disease (diabetes, rheumatoid arthritis),
- obesity,
- neurologic disease (Parkinson's disease, stroke)
- frailty and fallers.

Regarding ‘healthy’: *Healthy* older participants meet more criteria of healthy aging than pathological participants [15]; They fulfil the criterion free from disease and medication, i.e., no history of severe neurological, motor, cognitive, etc. disorders and medically stable (without unstable or acute medical condition, particularly those that prevent precluding daily activities or the exercise, any acute severe, rapidly progressive or terminal illness).

**Co-morbidities.** Summary of the reported co-morbidities.

**Overall health and activity assessment.** Reported physical, mental and activity assessments which refers to level of physical activity, functional fitness and mental state confirmed by tests, scores, or self-reported indexes (for example, Timed Up and Go (TUG), physical fall risk (PPA), Mini Mental State Evaluation, Montreal Cognitive Assessment, Attention, Attention Network Test Reaction Time, MOS SF-36).

**Technology-use experience.** Was there any form of technology experience inquired?

- availability of technology at home (yes, no),
- previous experience with technology (yes, no),
- types of device and services that was previously used: computer/notebook/laptop/ tablet, mobile phone/smartphone, smartwatch, internet, digital experience (digital games, digital photography, electronic books, ATMs, etc.), mail services, etc.

**2) INTERVENTION CHARACTERISTICS**

**Participant Recruitment and Selection.** Refers to the criteria for inclusion and exclusion based on health conditions, types of diseases, or other factors. E.g., cardiovascular disease primary or secondary prevention (e.g., hypertension, stroke, pacemakers), renal disease (e.g. kidney damage), respiratory diseases (e.g., chronic obstructive pulmonary disease), hearing or vision problems, independence in daily living, language proficiency, etc.

**Participant allocation.** Refers to how the researchers decide who receives what treatment in an experiment. There are three ways to allocate participants:

- randomly,
- non-randomly,
- no allocation.

**Tailoring and supervision.**

Supervision was categorized as:

- supervised (training under professional or non-professional supervision),
- unsupervised (home-based training alone),

Tailoring, customization, personalization of the intervention was categorized as:

- tailored - interventions that were customized or personalized to suit the specific needs or characteristics of individuals or groups of participants,
- generic intervention approach - implies a standardized or one-size-fits-all approach that was applied uniformly to all participants without customization.

**Activity characteristics.** Description of physical activity, duration, session frequency, and session length follows the PARS taxonomy [16]. While elements of the Consensus on Exercise Reporting Template [17] were considered, particularly those relevant to technology-based exercise programs, the full CERT checklist was not applied, as it was beyond the scope of this review.

**Session** characteristics for the intervention group, this refers to:

- intervention time frame/duration (the number of weeks of sessions),
- session frequency (the number of sessions per week),
- session length (possibly session time), and
- exercise intensity.

For comparator group: Refers to various factors, including: the absence of treatment, a waitlist, the usual care routine, treatment as usual, the standard of care, alternative interventions, modality, content, or detailed description of the type of exercise or physical activity.

**Purpose of physical activity.** Refers to specific physical, mental or activity domains which the intervention aims to improve. Multiple options are possible. The aims of the activities were categorized into the following domains: Improvement of …

- cardiorespiratory (endurance),
- muscular strength and power,
- flexibility,
- balance,
- neuromotor (neuromuscular), including motor skills, proprioception.
- functional mobility,
- physical activity level (general active lifestyle),
- mental and cognitive function, where cognitive functioning encompasses a wide range of mental processes essential for daily life, while mental functioning includes emotional and psychological dimensions.
- falls (risk) reduction.

**(3) TECHNOLOGY CHARACTERISTICS**

**Technology design.**

Hardware and software were categorized into (multiple types possible within one study):

- computer-based,
- sound and video recording systems (including telephone (e.g., voice-devices) and video recordings) for demonstration (pre-recorded) and feedback (live recording of participant) purposes,
- apps (e.g., web-based, tablet, or mobile-smart phone apps, telepresence programs, etc.),
- web video /phone call (call with person to check on/feedback correct application of technology, but also to ensure correct activity level),
- exergames (exercise in a virtual environment; including television, computer, smart phone, digital video games, screen, etc.,) [18],
- trackers (physical activity/ behavioural trackers, no feedback directly from the application (even if data is streamed back into app)),
- virtual reality (head-worn virtual reality immersive environment – example meta, google glasses, virtual reality can create immersive environments that make physical activity more engaging and enjoyable for elderly users),
- augmented reality, (using “glasses” to superimpose digital information on the real world – identification of correct exercises, form, intensity, trip hazards, etc, augmented reality can provide visual cues and feedback during physical activities, making it easier for elderly users to follow instructions and perform exercises correctly),
- wearable devices (e.g., Inertial Measurement Unit, can stream info back to the application, can provide real-time feedback on physical activity levels and other vital signs, making it easier for elderly users to track their progress and stay motivated),
- and telehealth systems (including telemedicine, tele-rehabilitation, etc.),
- others (e.g., step mats, digital elastic bands).

**Function and usability.**

The functions of digital interventions were **c**ategorized into (multiple functions possible):

- assessment of outcomes, (measuring diagnosis, decline of health-related / movement outcomes, etc),
- providing physical activity information (once by e.g., booklet, or video recordings) (to promote home-based exercise and physical activity etc.),
- providing physical activity information continuously (during the exercise/ intervention e.g., in an exergame),
- feedback of performance (feedback based on actual performance, regardless of purpose of improving activity or motivation, which can be visual, oral or a quantitative number),
- real-time health metrics (heart rate, movement intensity),
- real-time movement data (on the spot, high density data streaming, recording used for digital feedback, exergaming. e.g., camera registration the body movement),
- real-time functional ability (physical activity intensity, volume, gait quality, balance performance score, etc. clinical related outcome)
- other.

**Interaction with participant.**

Interfaces were categorized into (multiple interfaces possible):

- graphical user interface (user sees/uses graphics, e.g. graph and avatar, cartoon; video),
- command line interface (cli) (user reads/writes text) with feedback,
- command line written / typed information without feedback (, e.g. presenting the number of exercise repetitions),
- (haptic) touch user interface (directly on screen or pushing button as part of intervention (gloves, tactile sensation) and not to control e.g. the menu-driven interface),
- auditory/visual user (to explain or display e.g. an exercise description, or during a call or while providing feedback),
- menu-driven user interface (the menu-driven interface employs a series of screens, or “menus”. When a user selects by tapping/clicking on the list format or graphics, it takes them to the next menu screen until they complete the desired outcome),
- whole body movement interface (e.g., to drive the virtual reality app captured via video camera or gloves, activate the wearable devices, also whole-body movement influencing the cop on e.g., Wii balance board),
- form-based user interface (text box – drop-down menus, etc.),
- natural language processing user interface (auto-translator),
- other.

**(3) TECHNOLOGY AND HEALTH OUTCOMES**

**Outcomes focusing on Technology.** Refers to:

- adherence,
- adverse effects,
- dropouts,

and usability assessment (of effectiveness, efficiency, learnability, safety, accessibility, and satisfaction):

- Based on participant’s experience/post-study interview or questionnaires.
- Including: rating of technology use, general comments, intention to keep using or use the technology, enjoyment of using the technology,
- Participants' comments on improvement of the technology (design, accessibility),
- Outcomes of objective outcomes around experience (System Usability Score or Diagnostic Assessment Research Tool outcomes),
- Barriers and facilitators (any mentioned), etc.

**Outcomes focusing on Health.** Health outcomes were categorized into same classes as physical activity purposes. An additional category for Quality of Life outcome measures was added.

- cardiorespiratory (fitness, aerobic or cardiorespiratory endurance),
- muscular strength and power,
- flexibility (joint ROM),
- balance (Berg Balance Scale, CoP parameters, sway parameters, functional reach test etc.),
- neuromotor (neuromuscular); including motor skills, proprioception functional mobility (gait performance, walking parameters, Timed-up and Go test),
- physical activity level (International Physical Activity Questionnaire (IPAQ), number of steps, etc),
- mental and cognitive (executive, memory function),
- falls reduction (number of falls, fall risk, fear of falling),
- Quality of Life (e.g., SF36).

**REFERENCES**

1. Aggarwal R, Ranganathan P. Study designs: Part 4 - Interventional studies. Perspectives in clinical research 2019;10(3):137–139. PMID:31404185

2. National institute for Health and Care Research. Guidance on applying for feasibility studies. 2021. Available from: https://www.nihr.ac.uk/documents/guidance-on-applying-for-feasibility-studies/20474?pr=

3. Busetto L, Wick W, Gumbinger C. How to use and assess qualitative research methods. Neurological Research and Practice 2020 Dec 27;2(1):14. doi: 10.1186/s42466-020-00059-z

4. Ranganathan P, Aggarwal R. Study designs: Part 1 - An overview and classification. Perspectives in clinical research 2018;9(4):184–186. PMID:30319950

5. Aggarwal R, Ranganathan P. Study designs: Part 2 - Descriptive studies. Perspectives in clinical research 2019;10(1):34–36. PMID:30834206

6. Alterovitz SSR, Mendelsohn GA. Relationship goals of middle-aged, young-old, and old-old internet daters: An analysis of online personal ads. Journal of Aging Studies 2013 Apr;27(2):159–165. doi: 10.1016/j.jaging.2012.12.006

7. Kowal P, Dowd JE. Definition of an Older Person. Proposed Working Definition of an Older Person in Africa for the MDS Project. Geneva, Switzerland: World Health Organization; 2001. doi: 10.13140/2.1.5188.9286

8. Cohen-Mansfield J, Shmotkin D, Blumstein Z, Shorek A, Eyal N, Hazan H. The Old, Old-Old, and the Oldest Old: Continuation or Distinct Categories? An Examination of the Relationship between Age and Changes in Health, Function, and Wellbeing. The International Journal of Aging and Human Development 2013 Jul 19;77(1):37–57. doi: 10.2190/AG.77.1.c

9. U.S. Department of the interior. Standards for maintaining, collecting, and presenting federal data on race and ethnicity. 1997. Available from: https://www.doi.gov/pmb/eeo/directives/race-data

10. National Center for Health statistics. Race. 2023. Available from: https://www.cdc.gov/nchs/hus/sources-definitions/race.htm#print

11. European Commission. National Education Systems. 2024. Available from: https://eurydice.eacea.ec.europa.eu/national-education-systems

12. United Nations Educational Scientific and Cultural Organization. ISCED 1997. International standard classification of education. UNESCO Institute for Statistics; 2006. ISBN:92-9189-035-9

13. United Nations Educational Scientific and Cultural Organization. ISCED 2011. International standard classification of education. UNESCO Institute for Statistics; 2012.

14. World Health Organization. International Statistical Classification of Diseases and Related Health Problems (ICD). 2022. Available from: https://www.who.int/standards/classifications/classification-of-diseases

15. Menassa M, Stronks K, Khatami F, Roa Díaz ZM, Espinola OP, Gamba M, Itodo OA, Buttia C, Wehrli F, Minder B, Velarde MR, Franco OH. Concepts and definitions of healthy ageing: a systematic review and synthesis of theoretical models. eClinicalMedicine 2023 Feb;56:101821. doi: 10.1016/j.eclinm.2022.101821

16. Hanson CL, Oliver EJ, Dodd-Reynolds CJ, Pearsons A, Kelly P. A modified Delphi study to gain consensus for a taxonomy to report and classify physical activity referral schemes (PARS). International Journal of Behavioral Nutrition and Physical Activity International Journal of Behavioral Nutrition and Physical Activity; 2020;17(1):1–11. PMID:33267840

17. Slade SC, Dionne CE, Underwood M, Buchbinder R. Consensus on Exercise Reporting Template (CERT): Explanation and Elaboration Statement. British Journal of Sports Medicine 2016;50(23):1428–1437. doi: 10.1136/bjsports-2016-096651

18. López-Nava IH, Rodriguez MD, García-Vázquez JP, Perez-Sanpablo AI, Quiñones-Urióstegui I, Meneses-Peñaloza A, Castillo V, Cuaya-Simbro G, Armenta JS, Martínez A, Sánchez W, Caro K, Martínez-García AI, Favela J. Current state and trends of the research in exergames for the elderly and their impact on health outcomes: a scoping review. J Ambient Intell Human Comput 2023 Aug;14(8):10977–11009. doi: 10.1007/s12652-022-04364-0
