# Supplementary material for: Technology-Assisted Physical Activity Interventions for Older People in Their Home-Based Environment: Scoping Review
Source: JMIR Aging. 2025 Sep 15;8:e65746. doi: 10.2196/65746 (PMC12516299; doi:10.2196/65746)
Supplement: Multimedia Appendix 4 [file aging-v8-e65746-s004.docx]

**APPENDIX 4. OVERVIEW RESULTS**

**CONTENTS**

Overview of the extracted results for:

1. Study design,
2. Participant characteristics,
3. Intervention characteristics,
4. Technology characteristics.

Crosstabulation results for:

1. Inter-technology characteristics,
2. Technology versus participant characteristics,
3. Technology versus intervention type.

References:

1. References to the 148 included sources.
2. **STUDY DESIGN**

| **Year of publication** | **N (%)** |
| --- | --- |
| **All articles** | **148 (100)** |
| 2003  2006  2008  2009  2010  2011  2012  2013  2014  2015  2016  2017  2018  2019  2020  2021  2022 | 1 (1%)  1 (1%)  1 (1%)  2 (1%)  4 (3%)  4 (3%)  3 (2%)  10 (7%)  6 (4%)  11 (7%)  10 (7%)  11 (7%)  22 (15%)  6 (4%)  19 (13%)  22 (15%)  15 (10%) |

| **World Region** | **N (%)** | **Country** | **N** |
| --- | --- | --- | --- |
| **North America** | 56 (38%) | USA | 49 |
|  |  | Canada | 7 |
| **Europe** | 42 (28%) | Netherlands | 8 |
|  |  | Germany | 6 |
|  |  | Italy | 5 |
|  |  | Sweden | 5 |
|  |  | Switzerland | 4 |
|  |  | Denmark | 3 |
|  |  | UK | 3 |
|  |  | Belgium | 2 |
|  |  | Spain | 2 |
|  |  | Finland | 1 |
|  |  | France | 1 |
|  |  | Poland | 1 |
|  |  | Iceland | 1 |
| **Australia** | 17 (11%) | Australia | 16 |
|  |  | New Zealand | 1 |
| **Asia** | 18 (12%) | Korea  Taiwan  Hong Kong | 7  5  1 |
|  |  | China  Japan  India | 1  1  1 |
|  |  | Malaysia | 1 |
|  |  | Singapore | 1 |
| **Multiple** ^a^ | 7 (5%) |  | 7 |
| **Middle East/Eurasia** | 6 (4%) | Turkey | 1 |
|  |  | Iran | 2 |
|  |  | Israel  Russia | 2  1 |
| **South America** | 2 (1%) | Brazil | 2 |

^a^ Each study included 1) Germany, Spain, and Australia; 2) Sweden, Italy, Israel, The Netherlands, Germany, and USA; 3) Spain and Switzerland; 4) Australia, Germany, and Spain; 5) Australia and Canada; 6) Puerto Rico and USA; and 7) Belgium, Greece, UK, Switzerland, and The Netherlands.

| **Study design** | **N (%)** |
| --- | --- |
| Randomized Controlled Trials (RCT)  Pre- and post- test study  Feasibility study  Qualitative study  Experimental study  Non-randomized controlled trials  Mixed methods  Case study | 75 (51%)  35 (24%)  17 (12%)  6 (4%)  5 (3%)  5 (3%)  4 (2%)  1 (1%) |

1. **PARTICIPANT CHARACTERISTICS (SOCIO-DEMOGRAPHIC VALUES)**

| **Variable** | **Characteristics/ Number (nr of participants)** | **Articles,**  **N (%)** | **M (SD)** | **Range** |
| --- | --- | --- | --- | --- |
| **Participants**  nr= 12.717 | - | 147 (99%) | - | 1 – 1.650 |
| **Age, years**  nr= 12.462 |  | 144 (97%) | 74.0 (6.3) | 60.0 – 88.2 |
|  | Youngest-old, 60–74 years (nr= 9.802) | 87 (59%) | 69.9 (4.0) | - |
|  | Middle-old, 75–84 years (nr= 2.510) | 51 (35%) | 79.3 (2.8) | - |
|  | Oldest-old, ≥85 years (nr= 150) | 6 (4%) | 86.0 (1.2) | - |
| **Gender (% women)**  nr= 7.029 |  | 143 (97%) | 58.1 | 0–100 |
|  | Youngest-old, 60–74 years (nr=5.160) | 85 (57%) | 50.8 | - |
|  | Middle-old, 75–84 years (nr=1.750) | 50 (34%) | 68.3 | - |
|  | Oldest-old, ≥85 years (nr=119) | 6 (4%) | 77.8 | - |
| **Race**  nr= 4.545 | Reported in N (%) sources | 41 (28%) | - | - |
|  | Asian (nr= 63) | 2 (1%) | - | - |
|  | Hispanic/Latino (nr= 49) | 1 (1%) | - | - |
|  | White/Caucasian (nr= 267) | 9 (6%) | - | - |
|  | Mixed populations (nr= 4.166) | 29 (20%) | - | - |
|  | Non-White/Caucasian population in mixed studies, % | - | 27.9 (24%) | 2 – 95 |
|  | American Indian/Alaska Native | 2 (1%) | - | - |
|  | Asian | 4 (3%) | - | - |
|  | Black/African American | 10 (7%) | - | - |
|  | Hispanic/Latino | 4 (3%) | - | - |
|  | Multiracial | 1 (1%) | - | - |
|  | Native Hawaiian/Pacific Islander | 1 (1%) | - | - |
| **Income** |  |  |  |  |
| nr= 1.246 | Reported in N (%) sources | 13 (9%) | - | - |
|  |  |  |  |  |
|  | Low (nr= 70) | 2 (1%) | - | - |
|  | Mixed (nr= 1.176), within mixed: | 11 (7%) | - | - |
|  | Low-income population, % |  | 38.5 (19%) | 10.7 – 73.0 |
| **Education level**  nr= 7.279 | Reported in N (%) sources | 59 (40%) | 12.0 (3%) | 8 – 23 |
|  | Low/middle (nr= 947) | 11 (7%) | 9.1 (3%) | - |
|  | Middle/high (nr= 2.045) | 26 (18%) | 14.1 (2%) | - |
|  | Low/middle/high (nr= 4.287) | 22 (15%) | 10.7 (3%) | - |
| **Occupation**  nr= 2.292 |  |  |  |  |
|  | Retired (nr= 195) | 8 (5%) | - | - |
|  | Mixed population (nr= 2.097) | 13 (9%) | - | - |
|  | Performing job in mixed populations, % |  | 28.2 (18%) | 6.7–56.9 |
| **Residence**  nr= 12.717 |  |  |  |  |
|  | Care home (nr= 746) | 26 (18%) | - | - |
|  | Community dwelling (nr= 9.718) | 101 (68%) | - | - |
|  | Hospital (nr= 0) | 0 (0%) | - | - |
|  | Not defined (nr= 2.233) | 19 (13%) | - | - |
| **Population health status** |  |  |  |  |
| nr= 8.740 |  |  |  |  |
|  | Healthy (nr= 1.710) | 15 (10%) | - | - |
|  | Cancer (nr= 786) | 6 (4%) | - | - |
|  | Cardio-vascular disease (nr= 937) | 12 (8%) | - | - |
|  | Cognitive impairments (nr= 352) | 13 (9%) | - | - |
|  | COPD (chronic obstructive pulmonary disease) (nr= 632) | 4 (3%) | - | - |
|  | Diabetes (nr= 1.696) | 2 (1%) | - | - |
|  | Musculoskeletal disease (nr= 349) | 5 (3%) | - | - |
|  | Obesity (nr= 37) | 2 (1%) | - | - |
|  | Parkinson's disease (nr= 505) | 11 (7%) | - | - |
|  | Frailty (nr= 168) | 4 (3%) | - | - |
|  | Mixed population (nr= 1.568) | 20 (14%) | - | - |
| **Comorbidities** |  |  |  |  |
| nr= 5.948 |  | 55 (37%) | 4.2 (3%) | 1 – 10 |
|  |  |  |  |  |
| **Fallers vs non-fallers**  nr= 2.953 |  |  |  |  |
|  | Fallers (nr= 675) | 11 (7%) | - | - |
|  | Non-fallers (nr= 929) | 11 (7%) | - | - |
|  | Mixed (nr= 1.349) | 22 (15%) | - | - |
| **Overall health and activity assessment** |  | - | - | - |
|  | Physical assessment (nr= 5.463) | 80 (54%) | - | - |
|  | Mental assessment (nr= 2.654) | 49 (33%) | - | - |
|  | Activity assessment (nr= 6.317) | 55 (37%) | - | - |
| **Technology use assessment** |  |  |  |  |
|  | Presence at home (nr= 2.299) | 22 (15%) | - | - |
|  | Previous experience (nr= 2.402) | 24 (16%) | - | - |
| **Technology experience** | Devices and services | 24 (16%) | 2 | 1 – 4 |
|  | Computer/notebook/laptop/  Tablet (nr= 2.176) | 19 (13%) | - | - |
|  | Mobile phone/smartphone (nr= 802) | 11 (7%) | - | - |
|  | Smartwatch (nr= 242) | 1 (1%) | - | - |
|  | Internet (nr= 824) | 7 (5%) | - | - |
|  | Digital experience  (games, photography, books, ATMs, etc.) (nr= 23) | 2 (1%) | - | - |
|  | Email (nr= 368) | 1 (1%) | - | - |
|  | Availability of info’s about frequency of device/services usage (nr= 964) | 7 (5%) | - | - |

1. **INTERVENTION CHARACTERISTICS**

| **Tailoring, customisation, personalisation** | **Result** | **N (%)** | | |
| --- | --- | --- | --- | --- |
| Allocation | Randomly | 83 (56%) | | |
|  | Selectively | 12 (8%) | | |
|  | No allocation | 54 (36%) | | |
| Supervision | Professional | 67 (45%) | | |
|  | Non- professional | 81 (55%) | | |
| Personalisation | Generic | 55 (37%) | | |
|  | Tailored | 93 (63%) | | |
| Comparator (control group): | No Information | 6 (4%) | | |
|  | No treatment | | 18 (12%) |  |
|  | Wait list | | 8 (5%) |  |
|  | Usual care routine | | 21(14%) |  |
|  | Standard of care | | 3 (2%) |  |
|  | Alternative intervention | | 15 (10%) |  |
|  | Alternative modality | | 15 (10%) |  |
|  | Optimized care | | 2 (1%) |  |
|  | Alternative content | | 9 (6%) |  |
|  | No Comparator | | 52 (35%) |  |

| **Main intervention characteristics** | **Range and measurement type** | **N (%)** | **M (SD)** |
| --- | --- | --- | --- |
| Intervention duration (preferably weeks) | 1-4 | 16 (11%) | 2.4 (1.1) |
|  | 5-8 | 46 (31%) | 6.5 (1.1) |
|  | 9-12 | 46 (31%) | 10.3 (1.3) |
|  | 13-24 | 23 (16%) | 18.0 (3.3) |
|  | >25 | 16 (11%) | 123.7 (99.3) |
|  | n.i. | 4 (3%) | 0.0 |
| Frequency (sessions per week) | 1-2 | 33 (22%) | 1.5 (0.5) |
|  | 2.5-3 | 54 (37%) | 2.8 (0.2) |
|  | 4-7 | 27 (18%) | 5.3 (1.2) |
|  | 8-21 | 3 (2%) | 17.5 (3.5) |
|  | others | 2 (1%) | 0 |
|  | self-selected | 2 (1%) | n.i. |
|  | n.i. | 27 (18%) |  |
| Session duration (average min) | <15 | 9 (6%) | 9,4 (3.3) |
|  | 16-30 | 40 (27%) | 23,3 (4.3) |
|  | 31-45 | 22 (15%) | 38,1 (4.3) |
|  | 46-60 | 27 (18%) | 56,1 (3.4) |
|  | 61-75 | 4 (3%) | 69,2 (5.1) |
|  | 76-90 | 4 (3%) | 85,0 (5.0) |
|  | >91 | 2 (1%) | 120 (0.) |
|  | Highly variable  Self-selected  n.i. | 1 (1%)  3 (2%)  36 (24%) | 0  0  0 |
| Intensity | RPE scale | 5 (3%) | n.i. |
|  | Borg scale | 3 (2%) | n.i. |
|  | Four to five Tai Chi levels | 1 (1%) | n.i. |
|  | HRR of 60% | 1 (1%) | n.i. |
|  | Self-selected | 2 (1%) | n.i. |
|  | Others (level of the game)  n.i. | 9 (6%)  128 (86%) | n.i.  n.i. |
| Total sessions | 1-15 | 14 (10%) | 9,1 (2.9) |
|  | 16-30 | 47 (32%) | 23,0 (4.7) |
|  | 31-45 | 24 (16%) | 39,0 (4.6) |
|  | 46-60 | 14 (10%) | 54,0 (6.0) |
|  | 61-75 | 2 (1%) | 71,0 (1.0) |
|  | 76-100 | 7 (5%) | 86,0 (7.5) |
|  | >100 | 12 (8%) | 383,4 (549.2) |
|  | n.i. | 28 (19%) | n.i. |

n.i. = no information

| **Type of exercise and physical activity** | **Intervention group** | **Control Group** |
| --- | --- | --- |
|  | **N (%)** | **N (%)** |
| Cardiorespiratory Fitness | 30 (20%) | 6 (4%) |
| Muscular Strength/Power | 64 (43%) | 10 (7%) |
| Flexibility | 22 (15%) | 6 (4%) |
| Balance | 73 (49%) | 17 (12%) |
| Neuromotor including motor skills, proprioception | 19 (13%) | 1 (1%) |
| Functional mobility and physical performance | 28 (19%) | 4 (3%) |
| Physical Activity level | 25 (17%) | 6 (4%) |
| Mental, Cognitive | 23 (16%) | 3 (2%) |
| Fall reduction | 12 (8%) | 2 (1%) |
| No physical activity intervention for control group (They received: No treatment, Usual care routine, Optimized care) |  | 61 (41%) |

1. **TECHNOLOGY CHARACTERISTICS**

| **Variable** | **Categories** | **N (%)** |
| --- | --- | --- |
| **Design** | Computer-based  Sound and video recording systems  Mobile applications (web-based, tablet, mobile phone)  Web video/ phone call  Exergames (incl. screen)  Trackers  Virtual reality  Augmented reality  Wearable devices  Other | 20 (9%)  8 (4%)  48 (22%)  30 (13%)  68 (30%)  21 (9%)  6 (3%)  1 (0%)  14 (6%)  7 (3%) |
| **Function** | Assessment of outcomes  Providing exercise program information once  Providing exercise information continuously  Feedback of exercise  Real-time health metrics  Real-time movement data  Real-time functionality ability  Other | 21 (6%) 28 (8%)  102 (27%)  102 (27%)  6 (2%)  98 (26%)  9 (2%)  5 (1%) |
| **Interface** | Graphical interface  Command line with feedback  Command line without feedback  Menu-driven  (Haptic) touch  Auditory/ visual  Body movement  Form-based  Natural language processing  Remote driven  Other | 94 (27%) 24 (7%)  13 (4%)  40 (11%)  6 (2%) 61 (17%) 93 (27%) 1 (0%)  0 (0%)  14 (4%)  3 (1%) |

| **Variable** | **Characteristics/ Number (n)** | **Average (SD) or (%)** | **N (%)** |
| --- | --- | --- | --- |
| **Adverse events** | Reported – no  Reported – yes  None happened (N of sources, % of reported)  Grade 1 (mild, nr. of participants, % all adv events)  Grade 2 (moderate, nr. of participants)  Grade 3 (severe, nr. of participants)  **Technology related adverse events**  Grade 1 (mild, nr. of participants, % all adv events)) | 40 (51%)  10 (13%)  28 (36%)  2 (3%) | 80 (54%)  68 (46%)  49 (72%) |
| **Dropout** | Control group  Intervention groups | 13.9% (12%)  16.4% (16%) | 45 (30%)  100 (68%) |
| **Adherence level** | Control group  Intervention groups | 71.2% (25%)  80.0% (18%) | 16 (11%)  78 (53%) |

1. **TECHNOLOGY DESIGN * FUNCTION * INTERFACE**

| **DESIGN \ FUNCTION** | **Sum** | **Assess out-comes** | **One-time PA info** | **Continu PA Info** | **Feed back** | **Realtime health metrics** | **Realtime move-ment** | **Realtime funct. ability** | **other** |  |  |
| --- | --- | --- | --- | --- | --- | --- | --- | --- | --- | --- | --- |
| **Computer based (N=20)** | 48 | 10% | 10% | 27% | 25% | 2% | 25% |  |  |  |  |
| **Sound/video record. (N=8)** | 19 | 16% | 21% | 21% | 16% | 5% | 16% | 5% |  |  |  |
| **Mobile Apps (N=48)** | 102 | 10% | 15% | 29% | 20% | 2% | 19% | 4% | 2% |  |  |
| **Web video/phone (N=30)** | 63 | 6% | 11% | 27% | 32% | 2% | 17% | 5% |  |  |  |
| **Exergames (N=68)** | 205 | 4% | 2% | 31% | 29% | 1% | 31% | 1% | 0% |  |  |
| **Tracker (N=21)** | 48 | 8% | 6% | 15% | 31% | 2% | 31% | 6% |  |  |  |
| **Virtual reality (N=6)** | 23 | 4% | 22% | 22% | 17% | 26% | 4% | 4% |  |  |  |
| **Augmented reality (N=1)** | 3 |  |  | 33% | 33% |  | 33% |  |  |  |  |
| **Wearable device (N=14)** | 43 | 7% | 9% | 12% | 26% | 5% | 33% | 9% |  |  |  |
| **Other (N=7)** | 20 | 10% | 10% | 30% | 20% |  | 25% | 5% |  |  |  |
| **DESIGN \ INTERFACE** | **Sum** | **Graph-ical** | **Cmnd line w feedback** | **Cmnd line w/o feedback** | **Menu driven** | **Haptic touch** | **Auditory Visual** | **Body move-ment** | **Form based** | **Remote driven** | **Other** |
| **Computer based (N=20)** | 39 | 28% | 3% | 8% | 15% | 3% | 13% | 26% |  | 3% | 3% |
| **Sound/video record. (N=8)** | 15 | 20% |  |  | 13% |  | **53%** | 13% |  |  |  |
| **Mobile Apps (N=48)** | 117 | 21% | 9% | 10% | 17% | 1% | 20% | 15% | 1% | 3% | 3% |
| **Web video/phone (N=30)** | 72 | 24% | 8% | 6% | 15% | 3% | 26% | 17% |  |  | 1% |
| **Exergames (N=68)** | 178 | 35% | 4% | 1% | 10% | 1% | 11% | 32% |  | 7% |  |
| **Tracker (N=21)** | 52 | 25% | 13% | 4% | 8% | 2% | 13% | 29% | 2% | 2% | 2% |
| **Virtual reality (N=6)** | 12 | 25% |  |  |  |  | 33% | **42%** |  |  |  |
| **Augmented reality (N=1)** | 2 | 50% |  |  |  |  |  | 50% |  |  |  |
| **Wearable device (N=14)** | 37 | 19% | 19% |  | 8% | 3% | 14% | 38% |  |  |  |
| **Other (N=7)** | 14 | 29% | 14% |  | 21% | 7% | 7% | 21% |  |  |  |
| **FUNCTION \ INTERFACE** | **Sum** | **Graph-ical** | **Cmnd line w feedback** | **Cmnd line w/o feedback** | **Menu driven** | **Haptic touch** | **Auditory Visual** | **Body move-ment** | **Form based** | **Remote driven** | **Other** |
| **Assess outcomes (N=21)** | 49 | 31% | 10% | 2% | 12% |  | 12% | 22% |  | 8% | 2% |
| **One-time PA info (N=28)** | 66 | 18% | 11% | 9% | 17% | 5% | 20% | 14% | 2% | 3% | 3% |
| **Continu PA Info (N=102)** | 251 | 30% | 5% | 3% | 13% | 1% | 16% | 27% |  | 5% | 0% |
| **Feedback (N=102)** | 267 | 28% | 9% |  | 10% | 2% | 16% | 29% |  | 5% |  |
| **Realtime health metrics (N=6)** | 15 | 27% | 27% |  | 0% |  | 13% | 33% |  |  |  |
| **Realtime movement (N=98)** | 237 | 32% | 8% |  | 11% |  | 14% | 36% |  |  |  |
| **Realtime funct. ability (N=9)** | 25 | 24% | 20% |  | 12% | 4% | 12% | 24% |  | 4% |  |
| **Other (N=5)** | 9 | 11% | 0% | 11% | 11% | 11% | 22% | 33% |  |  |  |

In the left column, the design or function used in the study is listed with the total number of sources (N) in which this design or function is used. The sum in the second column represents the total number of different functions or interfaces used for each design or function respectively. The other columns represent the percentages of the sum, and the total of each row equals 100%. With abbreviations: Apps, applications; cmnd, command; exc, exercise; funct., functional; info, information; N, total number of sources; PA, physical activity; record., recording; w, with; w/o, without.

1. **TECHNOLOGY * POPULATION (age, health status, residency)**

| **DESIGN** | **Sum*** | **Com-puter based** | **Sound/ video record. systems** | **Mobile Apps** | **Web/ video phone call** | **Exer- games** | **Track-ers** | **Virtual reality** | **Aug-mented reality** | **Wear-able devices** | **Other** |
| --- | --- | --- | --- | --- | --- | --- | --- | --- | --- | --- | --- |
| Youngest old (65-74 y) | 125 | 7% | 2% | 24% | 15% | 25% | 14% | 2% |  | 6% | 4% |
| Middle old (75-85 y) | 75 | 11% | 7% | 21% | 13% | 33% | 3% | 4% |  | 7% | 1% |
| Oldest old (> 85+ y) | 18 | 11% | 6% | 11% | 6% | **50%** | 6% |  |  | 6% | 6% |
| Mixed | 3 |  |  |  |  | 67% |  |  | 33% |  |  |
| **FUNCTION** | **Sum** | **Assess out-comes** | **One time PA info** | **Continu PA info** | **Feed-back** | **Real-time health metrics** | **Real-time move-ment** | **Real-time funct. ability** | **Other** |  |  |
| Youngest old (65-74 y) | 198 | 6% | 9% | 25% | 27% | 2% | 27% | 4% | 1% |  |  |
| Middle old (75-85 y) | 128 | 5% | 6% | 31% | 28% | 1% | 25% | 2% | 2% |  |  |
| Oldest old (> 85+ y) | 36 | 8% | 6% | 28% | 25% | 3% | 31% | 0% | 0% |  |  |
| Mixed | 6 |  |  | 33% | 33% |  | 33% |  |  |  |  |
| **INTERFACE** | **Sum** | **Graph-ical** | **Cmnd w feed-back** | **Cmnd w/o feed-back** | **Menu driven** | **Haptic touch** | **Auditory Visual** | **Body move-ment** | **Form based** | **Remote driven** | **Other** |
| Youngest old (65-74 y) | 195 | 25% | 8% | 4% | 12% | 1% | 18% | 28% | 1% | 3% | 1% |
| Middle old (75-85 y) | 117 | 29% | 4% | 4% | 13% | 3% | 18% | 24% |  | 4% | 1% |
| Oldest old (> 85+ y) | 30 | 30% | 13% |  | 3% | 3% | 10% | 30% |  | 10% |  |
| Mixed | 4 | 50% |  |  |  |  |  | 25% |  | 25% |  |

| **DESIGN** | **Sum** | **Com-puter based** | **Sound/ video record. Systems** | **Mobile Apps** | **Web/ video phone call** | **Exer- games** | **Track-ers** | **Virtual reality** | **Aug-mented reality** | **Wear-able device** | **Other** |
| --- | --- | --- | --- | --- | --- | --- | --- | --- | --- | --- | --- |
| Healthy | 19 |  |  | 21% | 5% | 53% | 11% | 5% |  |  | 5% |
| Condition | 127 | 11% | 5% | 19% | 15% | 27% | 11% | 2% |  | 8% | 3% |
| **FUNCTION** | **Sum** | **Assess out-comes** | **One time PA info** | **Continu PA info** | **Feed-back** | **Real-time health metrics** | **Real-time move-ment** | **Real-time funct. ability** | **Other** |  |  |
| Healthy | 36 |  | 3% | 33% | 33% |  | 28% | 3% |  |  |  |
| Condition | 206 | 8% | 8% | 25% | 27% | 2% | 27% | 3% |  |  |  |
| **INTERFACE** | **Sum** | **Graph-ical** | **Cmnd w feed-back** | **Cmnd w/o feed-back** | **Menu driven** | **Haptic touch** | **Audi-tory Visual** | **Body move-ment** | **Form based** | **Re-mote driven** | **Other** |
| Healthy | 35 | 34% | 9% |  | 6% |  | 14% | 31% |  | 3% | 3% |
| Condition | 184 | 26% | 6% | 3% | 9% | 1% | 21% | 28% |  | 5% | 1% |

| **DESIGN** | **Sum** | **Com-puter based** | **Sound/ video record. Systems** | **Mobile Apps** | **Web/ video phone call** | **Exer- games** | **Track-ers** | **Virtual reality** | **Aug-mented reality** | **Wear-able devices** | **Other** |
| --- | --- | --- | --- | --- | --- | --- | --- | --- | --- | --- | --- |
| Care Homes | 42 | 10% | 5% | 7% | 2% | 50% | 7% | 10% |  | 2% | 7% |
| Community Dwelling | 154 | 9% | 3% | 24% | 18% | 26% | 10% | 1% | 1% | 5% | 3% |
| Other | 33 | 6% | 3% | 24% | 6% | 21% | 3% | 18% |  | 18% |  |
| **FUNCTION** | **Sum** | **Assess out-comes** | **One time PA info** | **Continu PA info** | **Feed-back** | **Real-time health metrics** | **Real-time move-ment** | **Real-time funct. ability** | **Other** |  |  |
| Care Homes | 79 | 6% | 1% | 30% | 28% | 1% | 32% |  | 1% |  |  |
| Community Dwelling | 232 | 4% | 9% | 29% | 28% | 1% | 25% | 2% | 1% |  |  |
| Other | 59 | 10% | 10% | 20% | 22% | 3% | 25% | 8% |  |  |  |
| **INTERFACE** | **Sum** | **Graph-ical** | **Cmnd w feed-back** | **Cmnd w/o feed-back** | **Menu driven** | **Haptic touch** | **Audi-tory Visual** | **Body move-ment** | **Form based** | **Remote driven** | **Other** |
| Care Homes | 61 | 31% | 2% |  | 7% | 2% | 20% | 34% |  | 5% | 5% |
| Community Dwelling | 236 | 26% | 7% | 5% | 13% | 2% | 19% | 25% | 0% | 3% | 1% |
| Other | 52 | 25% | 13% | 2% | 12% | 2% | 10% | 27% |  | 10% | 10% |

*The sum in the second column represents how often a specific exercise type was implemented using technology design, function or interface. The other columns represent the percentages of the sum, and the total of each row equals 100%. With abbreviations: Apps, applications; cmnd, command; exc, exercise; funct., functional; info, information; N, total number of sources; PA, physical activity; record., recording; w, with; w/o, without.

1. **TECHNOLOGY * INTERVENTION (type, tailoring)**

| **EXERCISE / DESIGN** | **Sum*** | **Com-puter based** | **Sound/ video record. systems** | **Mobile Apps** | **Web/ video phone call** | **Exer- games** | **Track-ers** | **Virtual reality** | **Aug-mented reality** | **Wear-able devices** | **Other** |
| --- | --- | --- | --- | --- | --- | --- | --- | --- | --- | --- | --- |
| Cardiovascular | 41 | 7% | 2% | 27% | 7% | 41% | 10% | 2% |  | 2% |  |
| Muscle strength, power | 96 | 6% | 7% | 28% | 16% | 28% | 7% | 1% | 1% | 2% | 3% |
| Flexibility | 35 | 9% | 9% | 23% | 23% | 23% | 11% |  |  |  | 3% |
| Balance | 109 | 7% | 7% | 24% | 12% | 34% | 5% | 3% |  | 5% | 4% |
| Neuromotor | 29 | 17% | 3% | 10% | 10% | 48% |  | 3% |  |  | 7% |
| Functional mobility | 38 | 5% |  | 24% | 16% | 39% | 5% |  |  | 5% | 5% |
| Physical activity | 39 | 3% |  | 15% | 15% | 15% | 33% | 3% |  | 15% |  |
| Mental, Cognitive | 32 | 9% | 3% | 9% | 19% | 50% | 3% | 3% |  |  | 3% |
| Fall reduction | 14 | 7% |  | 14% | 7% | 57% | 7% |  |  |  | 7% |
| **EXERCSIE / FUNCTION** | **sum** | **Assess out-comes** | **One time PA info** | **Continu PA info** | **Feed-back** | **Real-time health metrics** | **Real-time move-ment** | **Real-time funct. ability** | **Other** |  |  |
| Cardiovascular | 72 | 8% | 10% | 28% | 26% |  | 28% |  |  |  |  |
| Muscle strength, power | 143 | 4% | 11% | 31% | 27% | 1% | 23% | 2% | 1% |  |  |
| Flexibility | 45 | 4% | 16% | 31% | 20% |  | 24% | 4% |  |  |  |
| Balance | 185 | 5% | 8% | 31% | 26% | 1% | 25% | 3% | 1% |  |  |
| Neuromotor | 51 | 8% | 2% | 31% | 25% | 2% | 31% |  |  |  |  |
| Functional mobility | 72 | 3% | 4% | 32% | 29% | 3% | 25% | 4% | 0% |  |  |
| Physical activity | 58 | 2% | 7% | 16% | 34% | 3% | 34% | 2% | 2% |  |  |
| Mental, Cognitive | 62 | 5% | 3% | 31% | 27% | 2% | 31% |  | 2% |  |  |
| Fall reduction | 29 |  | 3% | 41% | 24% |  | 31% |  |  |  |  |
| **EXERCISE / INTERFACE** | **sum** | **Graph-ical** | **Cmnd w feed-back** | **Cmnd w/o feed-back** | **Menu driven** | **Haptic touch** | **Auditory Visual** | **Body move-ment** | **Form based** | **Remote driven** | **Other** |
| Cardiovascular | 75 | 27% | 5% | 4% | 7% |  | 20% | 25% | 1% | 9% | 1% |
| Muscle strength, power | 149 | 28% | 5% | 6% | 13% | 1% | 21% | 21% |  | 5% | 1% |
| Flexibility | 44 | 30% | 5% | 5% | 11% | 2% | 23% | 20% |  | 5% |  |
| Balance | 179 | 28% | 6% | 3% | 12% | 1% | 20% | 25% |  | 4% | 1% |
| Neuromotor | 36 | 28% |  | 3% | 6% | 3% | 22% | 36% |  | 3% |  |
| Functional mobility | 73 | 27% | 5% | 8% | 18% |  | 15% | 26% |  |  |  |
| Physical activity | 59 | 25% | 15% | 3% | 2% | 2% | 15% | 34% |  | 2% | 2% |
| Mental, Cognitive | 51 | 33% |  | 2% | 12% | 2% | 18% | 29% |  | 4% |  |
| Fall reduction | 32 | 28% | 3% |  | 22% | 3% | 13% | 31% |  |  |  |

| **DESIGN (% of N)** | **Sum** | **Com-puter based** | **Sound/ video record. systems** | **Mobile Apps** | **Web/ video phone call** | **Exer- games** | **Track-ers** | **Virtual reality** | **Aug-mented reality** | **Wear-able devices** | **Other** |
| --- | --- | --- | --- | --- | --- | --- | --- | --- | --- | --- | --- |
| Tailored | 144 | 9% | 3% | 26% | 15% | 27% | 8% | 3% | 1% | 6% | 3% |
| Not tailored | 80 | 9% | 5% | 14% | 11% | 36% | 11% | 3% | 1% | 6% | 4% |
| **FUNCTION (% of N)** | **Sum** | **Assess out-comes** | **One time PA info** | **Continu PA info** | **Feed-back** | **Real-time health metrics** | **Real-time move-ment** | **Real-time funct. ability** | **Other** |  |  |
| Tailored | 231 | 6% | 8% | 27% | 26% | 2% | 26% | 3% | 1% |  |  |
| Not tailored | 139 | 5% | 6% | 28% | 29% | 1% | 28% | 1% | 1% |  |  |
| **INTERFACE (% of N)** | **Sum** | **Graph-ical** | **Cmnd w feed-back** | **Cmnd w/o feed-back** | **Menu driven** | **Haptic touch** | **Auditory Visual** | **Body move-ment** | **Form based** | **Remote driven** | **Other** |
| Tailored | 232 | 26% | 7% | 5% | 13% | 2% | 18% | 25% |  | 2% | 1% |
| Not tailored | 117 | 28% | 7% | 2% | 8% | 2% | 16% | 29% | 1% | 8% | 0% |

*The sum in the second column represents how often a specific exercise type was implemented using technology design, function or interface. The other columns represent the percentages of the sum, and the total of each row equals 100%. With abbreviations: Apps, applications; cmnd, command; exc, exercise; funct., functional; info, information; N, total number of sources; PA, physical activity; record., recording; w, with; w/o, without.

1. **REFERENCES TO 148 INCLUDED SOURCES**
2. Sheehy L, Sveistrup H, Knoefel F, Taillon-Hobson A, Martin T, Egan M, et al. The Use of Home-Based Nonimmersive Virtual Reality to Encourage Physical and Cognitive Exercise in People With Mild Cognitive Impairment: A Feasibility Study. J Aging Phys Act. 2022 Apr 1;30(2):297–307.
3. Lee BJ, Park YH, Lee JY, Kim SJ, Jang Y, Lee JI. Smartphone Application Versus Pedometer to Promote Physical Activity in Prostate Cancer Patients. Telemed e-Health. 2019 Dec 1;25(12):1231–6.
4. Taylor ME, Close JCT, Lord SR, Kurrle SE, Webster L, Savage R, et al. Pilot feasibility study of a home-based fall prevention exercise program (StandingTall) delivered through a tablet computer (iPad) in older people with dementia. Australas J Ageing. 2020 Sep 1;39(3):e278–87.
5. Leskinen T, Suorsa K, Tuominen M, Pulakka A, Pentti J, Löyttyniemi E, et al. The Effect of Consumer-based Activity Tracker Intervention on Physical Activity among Recent Retirees - An RCT Study. Med Sci Sports Exerc. 2021 Aug 1;53(8):1756–65.
6. Lauzé M, Martel DD, Agnoux A, Sirois MJ, Émond M, Daoust R, et al. Feasibility, Acceptability and Effects of a Home-Based Exercise Program Using a Gerontechnology on Physical Capacities After a Minor Injury in Community-Living Older Adults: A Pilot Study. J Nutr Heal Aging. 2018 Jan 1;22(1):16–25.
7. Hong J, Kim SW, Joo H, Kong HJ. Effects of smartphone mirroring-based telepresence exercise on body composition and physical function in obese older women. Aging Clin Exp Res. 2022 May 1;34(5):1113–21.
8. Nikitina S, Didino D, Baez M, Casati F. Feasibility of virtual tablet-based group exercise among older adults in Siberia: Findings from two pilot trials. JMIR mHealth uHealth. 2018 Feb 1;6(2).
9. Janssen S, Tange H, Arends R. A Preliminary Study on the Effectiveness of Exergame Nintendo “wii Fit Plus” on the Balance of Nursing Home Residents. Games Health J. 2013 Apr 1;2(2):89–95.
10. Talbot LA, Gaines JM, Huynh TN, Metter EJ. A Home‐Based Pedometer‐Driven Walking Program to Increase Physical Activity in Older Adults with Osteoarthritis of the Knee: A Preliminary Study. J Am Geriatr Soc [Internet]. 2003 Mar 20;51(3):387–92. Available from: https://agsjournals.onlinelibrary.wiley.com/doi/10.1046/j.1532-5415.2003.51113.x
11. Albores J, Marolda C, Haggerty M, Gerstenhaber B, Zuwallack R. The use of a home exercise program based on a computer system in patients with chronic obstructive pulmonary disease. J Cardiopulm Rehabil Prev. 2013 Jan;33(1):47–52.
12. Pettersson B, Wiklund M, Janols R, Lindgren H, Lundin-Olsson L, Skelton DA, et al. ’Managing pieces of a personal puzzle’-Older people’s experiences of self-management falls prevention exercise guided by a digital program or a booklet. BMC Geriatr. 2019 Feb 18;19(1).
13. Hawley-Hague H, Tacconi C, Mellone S, Martinez E, Ford C, Chiari L, et al. Smartphone apps to support falls rehabilitation exercise: App development and usability and acceptability study. JMIR mHealth uHealth. 2020 Sep 1;8(9).
14. Van Het Reve E, Silveira P, Daniel F, Casati F, De Bruin ED. Tablet-based strength-balance training to motivate and improve adherence to exercise in independently living older people: Part 2 of a phase ii preclinical exploratory trial. J Med Internet Res. 2014;16(6).
15. Zalecki T, Gorecka-Mazur A, Pietraszko W, Surowka AD, Novak P, Moskala M, et al. Visual feedback training using WII Fit improves balance in Parkinson’s disease. Folia Med Cracov [Internet]. 2013;53(1):65–78. Available from: http://www.ncbi.nlm.nih.gov/pubmed/24858332
16. Gschwind YJ, Schoene D, Lord SR, Ejupi A, Valenzuela T, Aal K, et al. The effect of sensor-based exercise at home on functional performance associated with fall risk in older people - A comparison of two exergame interventions. Eur Rev Aging Phys Act. 2015 Jan 1;12(1).
17. Demeyer H, Louvaris Z, Frei A, Rabinovich RA, De Jong C, Gimeno-Santos E, et al. Physical activity is increased by a 12-week semiautomated telecoaching programme in patients with COPD: A multicentre randomised controlled trial. Thorax. 2017 Jan 30;72(5):415–23.
18. Pettersson B, Janols R, Wiklund M, Lundin-Olsson L, Sandlund M. Older adults’ experiences of behavior change support in a digital fall prevention exercise program: Qualitative study framed by the self-determination theory. J Med Internet Res. 2021 Jul 1;23(7).
19. Muñoz J, Mehrabi S, Li Y, Basharat A, Middleton LE, Cao S, et al. Immersive Virtual Reality Exergames for Persons Living With Dementia: User-Centered Design Study as a Multistakeholder Team During the COVID-19 Pandemic. JMIR Serious Games. 2022 Jan 1;10(1).
20. Hong J, Kim J, Kim SW, Kong HJ. Effects of home-based tele-exercise on sarcopenia among community-dwelling elderly adults: Body composition and functional fitness. Exp Gerontol. 2017 Jan 1;87:33–9.
21. Neumann S, Meidert U, Barberà-Guillem R, Poveda-Puente R, Becker H. Effects of an Exergame Software for Older Adults on Fitness, Activities of Daily Living Performance, and Quality of Life. Games Health J. 2018 Oct 1;7(5):341–6.
22. Chao YY, Scherer YK, Wu YW, Lucke KT, Montgomery CA. The feasibility of an intervention combining self-efficacy theory and Wii Fit exergames in assisted living residents: A pilot study. Geriatr Nurs (Minneap). 2013 Sep;34(5):377–82.
23. Lee J, Jung D, Byun J, Lee M. Effects of a combined exercise program using an iPad for older adults. Healthc Inform Res. 2016 Apr 1;22(2):65–72.
24. Golla A, Müller T, Wohlfarth K, Jahn P, Mattukat K, Mau W. Home-based balance training using Wii FitTM: A pilot randomised controlled trial with mobile older stroke survivors. Pilot Feasibility Stud. 2018 Apr 25;4(1).
25. Espay AJ, Baram Y, Dwivedi AK, Shukla R, Gartner M, Gaines L, et al. At-home training with closed-loop augmented-reality cueing device for improving gait in patients with Parkinson disease. J Rehabil Res Dev [Internet]. 2010;47(6):573. Available from: http://www.rehab.research.va.gov/jour/10/476/pdf/espay.pdf
26. Jaarsma T, Klompstra L, Ben Gal T, Ben Avraham B, Boyne J, Bäck M, et al. Effects of exergaming on exercise capacity in patients with heart failure: results of an international multicentre randomized controlled trial. Eur J Heart Fail. 2021 Jan 1;23(1):114–24.
27. Rosenberg D, Depp CA, Vahia I V., Reichstadt J, Palmer BW, Kerr J, et al. Exergames for subsyndromal depression in older adults: A pilot study of a novel intervention. Am J Geriatr Psychiatry. 2010;18(3):221–6.
28. Vestergaard S, Kronborg C, Puggaard L. Home-based video exercise intervention for community-dwelling frail older women: a randomized controlled trial. Aging Clin Exp Res [Internet]. 2008 Oct 27;20(5):479–86. Available from: http://link.springer.com/10.1007/BF03325155
29. Li F, Harmer P, Fitzgerald K, Winters-Stone K. A cognitively enhanced online Tai Ji Quan training intervention for community-dwelling older adults with mild cognitive impairment: A feasibility trial. BMC Geriatr. 2022 Dec 1;22(1).
30. Davis JC, Liang Hsu C, Cheung W, Brasher PMA, Li LC, Khan KM, et al. Can the Otago falls prevention program be delivered by video? A feasibility study. BMJ open Sport Exerc Med [Internet]. 2016;e000059. Available from: http://dx.doi.org/10.1136/bmjsem-2015-000059
31. Fillol F, Paris L, Pascal S, Mulliez A, Roques CF, Rousset S, et al. Possible Impact of a 12-Month Web- And Smartphone-Based Program to Improve Long-term Physical Activity in Patients Attending Spa Therapy: Randomized Controlled Trial. J Med Internet Res. 2022 Jun 1;24(6).
32. Duarte-Rojo A, Bloomer PM, Rogers RJ, Hassan MA, Dunn MA, Tevar AD, et al. Introducing EL-FIT (Exercise and Liver FITness): A Smartphone App to Prehabilitate and Monitor Liver Transplant Candidates. Liver Transplant. 2021 Apr 1;27(4):502–12.
33. Zadro JR, Shirley D, Nilsen TIL, Mork PJ, Ferreira PH. Family History Influences the Effectiveness of Home Exercise in Older People With Chronic Low Back Pain: A Secondary Analysis of a Randomized Controlled Trial. Arch Phys Med Rehabil. 2020 Aug 1;101(8):1322–31.
34. Loh KP, Sanapala C, Watson EE, Jensen-Battaglia M, Janelsins MC, Klepin HD, et al. A single-arm pilot study of a mobile health exercise intervention (GO-EXCAP) in older patients with myeloid neoplasms. Blood Adv. 2022 Jul 12;6(13):3850–60.
35. Wu YZ, Lin JY, Wu PL, Kuo YF. Effects of a hybrid intervention combining exergaming and physical therapy among older adults in a long-term care facility. Geriatr Gerontol Int. 2019 Feb 1;19(2):147–52.
36. Yuan RY, Chen SC, Peng CW, Lin YN, Chang YT, Lai CH. Effects of interactive video-game-based exercise on balance in older adults with mild-to-moderate Parkinson’s disease. J Neuroeng Rehabil. 2020 Jul 13;17(1).
37. Duscha BD, Piner LW, Patel MP, Crawford LE, Jones WS, Patel MR, et al. Effects of a 12-Week mHealth Program on Functional Capacity and Physical Activity in Patients With Peripheral Artery Disease. Am J Cardiol. 2018 Sep 1;122(5):879–84.
38. Wang CH, Chou PC, Joa WC, Chen LF, Sheng TF, Ho SC, et al. Mobile-phone-based home exercise training program decreases systemic inflammation in COPD: A pilot study. BMC Pulm Med. 2014;14(1).
39. Auerswald T, Meyer J, von Holdt K, Voelcker-Rehage C. Application of activity trackers among nursing home residents—a pilot and feasibility study on physical activity behavior, usage behavior, acceptance, usability and motivational impact. Int J Environ Res Public Health. 2020 Sep 2;17(18):1–21.
40. Goode AP, Taylor SS, Hastings SN, Stanwyck C, Coffman CJ, Allen KD. Effects of a Home-Based Telephone-Supported Physical Activity Program for Older Adult Veterans With Chronic Low Back Pain. Phys Ther [Internet]. 2018 May 1;98(5):369–80. Available from: https://academic.oup.com/ptj/article/98/5/369/4925491
41. Schwartz H, Har-Nir I, Wenhoda T, Halperin I. Staying physically active during the COVID-19 quarantine: Exploring the feasibility of live, online, group training sessions among older adults. Transl Behav Med. 2021 Feb 1;11(2):314–22.
42. van den Helder J, Mehra S, van Dronkelaar C, ter Riet G, Tieland M, Visser B, et al. Blended home-based exercise and dietary protein in community-dwelling older adults: a cluster randomized controlled trial. J Cachexia Sarcopenia Muscle. 2020 Dec 1;11(6):1590–602.
43. Barnason S, Zimmerman L, Schulz P, Tu C. Influence of an early recovery telehealth intervention on physical activity and functioning after coronary artery bypass surgery among older adults with high disease burden. Hear Lung J Acute Crit Care. 2009 Nov;38(6):459–68.
44. McAuley E, Wójcicki TR, Gothe NP, Mailey EL, Szabo AN, Fanning J, et al. Effects of a DVD-delivered exercise intervention on physical function in older adults. Journals Gerontol - Ser A Biol Sci Med Sci. 2013 Sep;68(9):1076–82.
45. Bao T, Noohi F, Kinnaird C, Carender WJ, Barone VJ, Peethambaran G, et al. Retention Effects of Long-Term Balance Training with Vibrotactile Sensory Augmentation in Healthy Older Adults. Sensors. 2022 Apr 1;22(8).
46. Agmon M, Perry CK, Phelan E, Demiris G, Nguyen HQ. A pilot study of Wii Fit exergames to improve balance in older adults. J Geriatr Phys Ther. 2011 Oct;34(4):161–7.
47. Moy ML, Collins RJ, Martinez CH, Kadri R, Roman P, Holleman RG, et al. An Internet-Mediated Pedometer-Based Program Improves Health-Related Quality-of-Life Domains and Daily Step Counts in COPD. Chest [Internet]. 2015 Jul;148(1):128–37. Available from: http://journal.publications.chestnet.org/
48. Anderson-Hanley C, Arciero PJ, Brickman AM, Nimon JP, Okuma N, Westen SC, et al. Exergaming and older adult cognition: A cluster randomized clinical trial. Am J Prev Med. 2012 Feb;42(2):109–19.
49. Dekker-van Weering M, Jansen-Kosterink S, Frazer S, Vollenbroek-Hutten M. User experience, actual use, and effectiveness of an information communication technology-supported home exercise program for pre-frail older adults. Front Med. 2017;4.
50. Coultas DB, Jackson BE, Russo R, Peoples J, Singh KP, Sloan J, et al. Home-based physical activity coaching, physical activity, and health care utilization in chronic obstructive pulmonary disease chronic obstructive pulmonary disease self-management activation research trial secondary outcomes. Ann Am Thorac Soc. 2018 Apr 1;15(4):470–8.
51. Schwenk M, Grewal GS, Honarvar B, Schwenk S, Mohler J, Khalsa DS, et al. Interactive balance training integrating sensor-based visual feedback of movement performance: A pilot study in older adults. J Neuroeng Rehabil. 2014 Dec 13;11(1).
52. Esculier J, Vaudrin J, Bériault P, Gagnon K, Tremblay L. Home-based balance training programme using Wii Fit with balance board for Parkinsons´s disease: A pilot study. J Rehabil Med [Internet]. 2012;44(2):144–50. Available from: https://medicaljournalssweden.se/jrm/article/view/16003
53. Snyder A, Colvin B, Gammack JK. Pedometer Use Increases Daily Steps and Functional Status in Older Adults. J Am Med Dir Assoc. 2011;12(8):590–4.
54. Wu G, Keyes L, Callas P, Ren X, Bookchin B. Comparison of Telecommunication, Community, and Home-Based Tai Chi Exercise Programs on Compliance and Effectiveness in Elders at Risk for Falls. Arch Phys Med Rehabil. 2010 Jun;91(6):849–56.
55. Swinnen N, Vandenbulcke M, de Bruin ED, Akkerman R, Stubbs B, Vancampfort D. Exergaming for people with major neurocognitive disorder: a qualitative study. Disabil Rehabil. 2022;44(10):2044–52.
56. Garcia JA, Schoene D, Lord SR, Delbaere K, Valenzuela T, Navarro KF. A Bespoke Kinect Stepping Exergame for Improving Physical and Cognitive Function in Older People: A Pilot Study. Games Health J. 2016 Dec 1;5(6):382–8.
57. Olafsdottir SA, Jonsdottir H, Bjartmarz I, Magnusson C, Caltenco H, Kytö M, et al. Feasibility of ActivABLES to promote home-based exercise and physical activity of community-dwelling stroke survivors with support from caregivers: A mixed methods study. BMC Health Serv Res. 2020 Jun 22;20(1).
58. Villumsen BR, Jorgensen MG, Frystyk J, Hørdam B, Borre M. Home-based ‘exergaming’ was safe and significantly improved 6-min walking distance in patients with prostate cancer: a single-blinded randomised controlled trial. BJU Int. 2019 Oct 1;124(4):600–8.
59. Zahedian-Nasab N, Jaberi A, Shirazi F, Kavousipor S. Effect of virtual reality exercises on balance and fall in elderly people with fall risk: a randomized controlled trial. BMC Geriatr. 2021 Dec 1;21(1).
60. Klompstra L, Jaarsma T, Mårtensson J, Strömberg A. Exergaming Through the Eyes of Patients with Heart Failure: A Qualitative Content Analysis Study. Games Health J. 2017 Jun 1;6(3):152–8.
61. Schoene D, Lord SR, Delbaere K, Severino C, Davies TA, Smith ST. A Randomized Controlled Pilot Study of Home-Based Step Training in Older People Using Videogame Technology. PLoS One. 2013 Mar 5;8(3).
62. O’Brien T, Troutman-Jordan M, Hathaway D, Armstrong S, Moore M. Acceptability of wristband activity trackers among community dwelling older adults. Geriatr Nurs (Minneap). 2015 Mar 1;36(2):S21–5.
63. Wu G, Keyes LM. Group Tele-Exercise for Improving Balance in Elders. Telemed e-Health [Internet]. 2006 Oct;12(5):561–70. Available from: https://www.liebertpub.com/doi/10.1089/tmj.2006.12.561
64. Padala KP, Padala PR, Lensing SY, Dennis RA, Bopp MM, Roberson PK, et al. Home-Based Exercise Program Improves Balance and Fear of Falling in Community-Dwelling Older Adults with Mild Alzheimer’s Disease: A Pilot Study. J Alzheimer’s Dis. 2017;59(2):565–74.
65. Hosteng KR, Simmering JE, Polgreen LA, Cremer JF, Segre AM, Francis SL, et al. Multilevel mhealth intervention increases physical activity of older adults living in retirement community. J Phys Act Heal. 2021;18(7):851–7.
66. Golsteijn RHJ, Bolman C, Volders E, Peels DA, De Vries H, Lechner L. Short-term efficacy of a computer-tailored physical activity intervention for prostate and colorectal cancer patients and survivors: A randomized controlled trial. Int J Behav Nutr Phys Act. 2018 Oct 30;15(1).
67. Yousefi Babadi S, Daneshmandi H. Effects of virtual reality versus conventional balance training on balance of the elderly. Exp Gerontol. 2021 Oct 1;153.
68. Swinnen N, Vandenbulcke M, de Bruin ED, Akkerman R, Stubbs B, Firth J, et al. The efficacy of exergaming in people with major neurocognitive disorder residing in long-term care facilities: a pilot randomized controlled trial. Alzheimer’s Res Ther. 2021 Dec 1;13(1).
69. Jung DI, Ko DSi, Jeong M ae. Kinematic effect of Nintendo Wii TM sports program exercise on obstacle gait in elderly women with falling risk. J Phys Ther Sci. 2015;27(5).
70. Yeşilyaprak SS, Yildirim MŞ, Tomruk M, Ertekin Ö, Algun ZC. Comparison of the effects of virtual reality-based balance exercises and conventional exercises on balance and fall risk in older adults living in nursing homes in Turkey. Physiother Theory Pract. 2016 Apr 2;32(3):191–201.
71. Padala KP, Padala PR, Malloy TR, Geske JA, Dubbert PM, Dennis RA, et al. Wii-fit for improving gait and balance in an assisted living facility: A pilot study. Vol. 2012, Journal of Aging Research. 2012.
72. Maddison R, Pfaeffli L, Whittaker R, Stewart R, Kerr A, Jiang Y, et al. A mobile phone intervention increases physical activity in people with cardiovascular disease: Results from the HEART randomized controlled trial. Eur J Prev Cardiol. 2015 Jun 11;22(6):701–9.
73. van Diest M, Stegenga J, Wörtche HJ, Verkerke GJ, Postema K, Lamoth CJC. Exergames for unsupervised balance training at home: A pilot study in healthy older adults. Gait Posture. 2016 Feb 1;44:161–7.
74. Silveira P, Reve E van het, Daniel F, Casati F, De Bruin ED. Motivating and assisting physical exercise in independently living older adults: A pilot study. Int J Med Inform. 2013 May;82(5):325–34.
75. Li F, Harmer P, Voit J, Chou LS. Implementing an online virtual falls prevention intervention during a public health pandemic for older adults with mild cognitive impairment: A feasibility trial. Clin Interv Aging. 2021;16:973–83.
76. Far IK, Ferron M, Ibarra F, Baez M, Tranquillini S, Casati F, et al. The interplay of physical and social wellbeing in older adults: Investigating the relationship between physical training and social interactions with virtual social environments. PeerJ Comput Sci. 2015;2015(11).
77. Cinini A, Cutugno P, Ferraris C, Ferretti M, Marconi L, Morgavi G, et al. Final results of the NINFA project: impact of new technologies in the daily life of elderly people. Aging Clin Exp Res. 2021 May 1;33(5):1213–22.
78. McDermott MM, Spring B, Berger JS, Treat-Jacobson D, Conte MS, Creager MA, et al. Effect of a home-based exercise intervention of wearable technology and telephone coaching on walking performance in peripheral artery disease: The honor randomized clinical trial. JAMA - J Am Med Assoc. 2018 Apr 24;319(16):1665–76.
79. Song J, Paul SS, Caetano MJD, Smith S, Dibble LE, Love R, et al. Home-based step training using videogame technology in people with Parkinson’s disease: a single-blinded randomised controlled trial. Clin Rehabil. 2018 Mar 1;32(3):299–311.
80. Chao YY, Scherer YK, Montgomery CA, Wu YW, Lucke KT. Physical and Psychosocial Effects of Wii Fit Exergames Use in Assisted Living Residents: A Pilot Study. Clin Nurs Res. 2015 Dec 1;24(6):589–603.
81. Geraedts HAE, Zijlstra W, Zhang W, Spoorenberg SLW, Báez M, Far IK, et al. A home-based exercise program driven by tablet application and mobility monitoring for frail older adults: Feasibility and practical implications. Prev Chronic Dis. 2017;14:E12.
82. Pischke CR, Voelcker-Rehage C, Ratz T, Peters M, Buck C, Meyer J, et al. Web-Based Versus Print-Based Physical Activity Intervention for Community-Dwelling Older Adults: Crossover Randomized Trial. JMIR mHealth uHealth. 2022 Mar 1;10(3).
83. Batsis JA, Petersen CL, Clark MM, Cook SB, Kotz D, Gooding TL, et al. Feasibility and acceptability of a technology-based, rural weight management intervention in older adults with obesity. BMC Geriatr. 2021 Dec 1;21(1):44.
84. Gschwind YJ, Eichberg S, Ejupi A, de Rosario H, Kroll M, Marston HR, et al. ICT-based system to predict and prevent falls (iStoppFalls): Results from an international multicenter randomized controlled trial. Eur Rev Aging Phys Act. 2015 Jan 1;12(1).
85. Valenzuela T, Razee H, Schoene D, Lord SR, Delbaere K. An interactive home-based cognitive-motor step training program to reduce fall risk in older adults: Qualitative descriptive study of older adults’ experiences and requirements. JMIR Aging. 2018 Jul 1;1(2).
86. Jansons P, Dalla Via J, Daly RM, Fyfe JJ, Gvozdenko E, Scott D. Delivery of Home-Based Exercise Interventions in Older Adults Facilitated by Amazon Alexa: A 12-week Feasibility Trial. J Nutr Heal Aging. 2022 Jan 1;26(1):96–102.
87. Rodrigues EV, Gallo LH, Guimarães ATB, Melo Filho J, Luna BC, Gomes ARS. Effects of dance exergaming on depressive symptoms, fear of falling, and musculoskeletal function in fallers and nonfallers community-dwelling older women. Rejuvenation Res. 2018 Dec 1;21(6):518–26.
88. Morrison S, Simmons R, Colberg SR, Parson HK, Vinik AI. Supervised Balance Training and Wii Fit–Based Exercises Lower Falls Risk in Older Adults With Type 2 Diabetes. J Am Med Dir Assoc. 2018 Feb 1;19(2):185.e7-185.e13.
89. Muñoz GF, Cardenas RAM, Pla F. A kinect-based interactive system for home-assisted active aging. Sensors (Switzerland). 2021 Jan 2;21(2):1–26.
90. Brickwood KJ, Dk Ahuja K, Watson G, O’brien JA, Williams AD. The effects of Activity Tracker use with health professional support or Telephone Counselling on maintenance of physical activity and health outcomes in older adults: A Randomised Controlled Trial. JMIR Mhealth Uhealth [Internet]. 2021;9(1):e18686. Available from: https://doi.org/10.2196/preprints.18686
91. Peng X, Su Y, Hu Z, Sun X, Li X, Dolansky MA, et al. Home-based telehealth exercise training program in Chinese patients with heart failure A randomized controlled trial. Med (United States). 2018 Aug 1;97(35).
92. Mehra S, van den Helder J, Visser B, Engelbert RHH, Weijs PJM, Kröse BJA. Evaluation of a Blended Physical Activity Intervention for Older Adults: Mixed Methods Study. J Med Internet Res [Internet]. 2020 Jul 23;22(7):e16380. Available from: https://doi.org/10.2196/preprints.16380
93. Lewis ZH, Ottenbacher KJ, Fisher SR, Jennings K, Brown AF, Swartz MC, et al. The feasibility and RE-AIM evaluation of the TAME health pilot study. Int J Behav Nutr Phys Act. 2017 Aug 14;14(1).
94. Yin Z, Martinez CE, Li S, Martinez M, Peng K, Land WM, et al. Adapting Chinese Qigong mind-body exercise for healthy aging in older community-dwelling low-income latino adults: Pilot feasibility study. JMIR Aging. 2021 Oct 1;4(4).
95. Klompstra L, Jaarsma T, Strömberg A. Exergaming to increase the exercise capacity and daily physical activity in heart failure patients: A pilot study. BMC Geriatr. 2014;14(1).
96. Irvine AB, Gelatt VA, Seeley JR, Macfarlane P, Gau JM. Web-based intervention to promote physical activity by sedentary older adults: Randomized controlled trial. J Med Internet Res. 2013;15(2).
97. Mansson L, Lundin-Olsson L, Skelton DA, Janols R, Lindgren H, Rosendahl E, et al. Older adults’ preferences for, adherence to and experiences of two self-management falls prevention home exercise programmes: A comparison between a digital programme and a paper booklet. BMC Geriatr. 2020 Jun 15;20(1).
98. Anderson-Hanley C, Stark J, Wall KM, Vanbrakle M, Michel M, Maloney M, et al. The interactive physical and cognitive exercise system (iPACESxsTM): Effects of a 3-month in-home pilot clinical trial for mild cognitive impairment and caregivers. Clin Interv Aging. 2018;13:1565–77.
99. Martel D, Lauzé M, Agnoux A, Fruteau de Laclos L, Daoust R, Émond M, et al. Comparing the effects of a home-based exercise program using a gerontechnology to a community-based group exercise program on functional capacities in older adults after a minor injury. Exp Gerontol. 2018 Jul 15;108:41–7.
100. Chao Y, Scherer YK, Montgomery CA, Lucke KT, Wu YW. Exergames-based intervention for assisted living residents: a pilot study. Vol. 40, Journal of Gerontological Nursing. 2014.
101. Baez M, Far IK, Ibarra F, Ferron M, Didino D, Casati F. Effects of online group exercises for older adults on physical, psychological and social wellbeing: A randomized pilot trial. PeerJ. 2017;2017(4).
102. Auerswald T, Hendker A, Ratz T, Lippke S, Pischke CR, Peters M, et al. Impact of Activity Tracker Usage in Combination with a Physical Activity Intervention on Physical and Cognitive Parameters in Healthy Adults Aged 60+: A Randomized Controlled Trial. Int J Environ Res Public Health. 2022 Apr 1;19(7).
103. Netz Y, Yekutieli Z, Arnon M, Argov E, Tchelet K, Benmoha E, et al. Personalized Exercise Programs Based upon Remote Assessment of Motor Fitness: A Pilot Study among Healthy People Aged 65 Years and Older. Gerontology. 2022 May 1;68(4):465–79.
104. Gandolfi M, Geroin C, Dimitrova E, Boldrini P, Waldner A, Bonadiman S, et al. Virtual Reality Telerehabilitation for Postural Instability in Parkinson’s Disease: A Multicenter, Single-Blind, Randomized, Controlled Trial. Biomed Res Int. 2017;2017:1–11.
105. Ambrens M, Van Schooten KS, Lung T, Clemson L, Close JCT, Howard K, et al. Economic evaluation of the e-Health StandingTall balance exercise programme for fall prevention in people aged 70 years and over. Age Ageing. 2022 Jun 1;51(6).
106. Baez M, Ibarra F, Far IK, Ferron M, Casati F. Online Group-exercises for Older Adults of Different Physical Abilities. IEEE; 2016 Int Conf Collab Technol Syst [Internet]. 2016 Sep 17;524–33. Available from: http://arxiv.org/abs/1609.05329
107. Monteblanco Cavalcante M, Fraga I, Dalbosco B, De Marchi P, Iraci L, Baechtold da Silva ME, et al. Exergame training-induced neuroplasticity and cognitive improvement in institutionalized older adults: A preliminary investigation. Physiol Behav. 2021 Nov 1;241.
108. Wall K, Stark J, Schillaci A, Saulnier ET, McLaren E, Striegnitz K, et al. The enhanced interactive physical and cognitive exercise system (iPACESTM v2.0): Pilot clinical trial of an in-home iPad-based neuro-exergame for mild cognitive impairment (MCI). J Clin Med. 2018;7(9).
109. Shubert TE, Chokshi A, Mendes VM, Grier S, Buchanan H, Basnett J, et al. Stand Tall-A Virtual Translation of the Otago Exercise Program. J Geriatr Phys Ther. 2020 Jul 1;43(3):120–7.
110. Lynch BM, Nguyen NH, Moore MM, Reeves MM, Rosenberg DE, Boyle T, et al. A randomized controlled trial of a wearable technology-based intervention for increasing moderate to vigorous physical activity and reducing sedentary behavior in breast cancer survivors: The ACTIVATE Trial. Cancer. 2019 Aug 15;125(16):2846–55.
111. Yang WC, Wang HK, Wu RM, Lo CS, Lin KH. Home-based virtual reality balance training and conventional balance training in Parkinson’s disease: A randomized controlled trial. J Formos Med Assoc. 2016 Sep 1;115(9):734–43.
112. Kim T, Xiong S. Effectiveness and Usability of a Novel Kinect-Based Tailored Interactive Fall Intervention System for Fall Prevention in Older People: A Preliminary Study. Front Public Heal. 2022 May 31;10.
113. Callisaya ML, Jayakody O, Vaidya A, Srikanth V, Farrow M, Delbaere K. A novel cognitive-motor exercise program delivered via a tablet to improve mobility in older people with cognitive impairment – StandingTall Cognition and Mobility. Exp Gerontol. 2021 Sep 1;152.
114. Sparrow D, Gottlieb DJ, Demolles D, Fielding RA. Increases in muscle strength and balance using a resistance training program administered via a telecommunications system in older adults. Journals Gerontol - Ser A Biol Sci Med Sci. 2011 Nov;66(11):1251–7.
115. Jennings SC, Manning KM, Bettger JP, Hall KM, Pearson M, Mateas C, et al. Rapid Transition to Telehealth Group Exercise and Functional Assessments in Response to COVID-19. Gerontol Geriatr Med. 2020;6.
116. Flynn A, Preston E, Dennis S, Canning CG, Allen NE. Home-based exercise monitored with telehealth is feasible and acceptable compared to centre-based exercise in Parkinson’s disease: A randomised pilot study. Clin Rehabil. 2021 May 1;35(5):728–39.
117. Szanton SL, Walker RK, Lim JH, Fisher L, Zhan A, Gitlin LN, et al. Development of an exergame for Urban-dwelling older adults with functional limitations: Results and lessons learned. Prog Community Heal Partnerships Res Educ Action. 2016 Mar 1;10(1):73–81.
118. Alagumoorthi G, Beulah Jebakani D, Thirunavukarasu S, Ramachandaran V, Kumaresan A. Effectiveness of Wii sports- based strategy training in reducing risk of falling, falls and improving quality of life in adults with idiopathic Parkinson’s disease- a randomized comparative trial. Clin Rehabil. 2022 Aug 1;36(8):1097–109.
119. Hong J, Kong HJ, Yoon HJ. Web-based telepresence exercise program for community-dwelling elderly women with a high risk of falling: Randomized controlled trial. JMIR mHealth uHealth. 2018 May 1;6(5).
120. Muellmann S, Buck C, Voelcker-Rehage C, Bragina I, Lippke S, Meyer J, et al. Effects of two web-based interventions promoting physical activity among older adults compared to a delayed intervention control group in Northwestern Germany: Results of the PROMOTE community-based intervention trial. Prev Med Reports. 2019 Sep 1;15.
121. Ellmers TJ, Paraskevopoulos IT, Williams AM, Young WR. Recalibrating disparities in perceived and actual balance abilities in older adults: A mixed-methods evaluation of a novel exergaming intervention. J Neuroeng Rehabil. 2018 Mar 22;15(1).
122. Ogawa EF, Huang H, Yu LF, Gona PN, Fleming RK, Leveille SG, et al. Effects of Exergaming on Cognition and Gait in Older Adults at Risk for Falling. Med Sci Sports Exerc. 2020 Mar 1;52(3):754–61.
123. Daly RM, Gianoudis J, Hall T, Mundell NL, Maddison R. Feasibility, usability, and enjoyment of a home-based exercise program delivered via an exercise app for musculoskeletal health in community-dwelling older adults: Short-term prospective pilot study. Vol. 9, JMIR mHealth and uHealth. JMIR Publications Inc.; 2021. p. e21094.
124. Soancatl Aguilar V, Lamoth CJC, Maurits NM, Roerdink JBTM. Assessing dynamic postural control during exergaming in older adults: A probabilistic approach. Gait Posture. 2018 Feb 1;60:235–40.
125. Batsis JA, Naslund JA, Gill LE, Masutani RK, Agarwal N, Bartels SJ. Use of a Wearable Activity Device in Rural Older Obese Adults. Gerontol Geriatr Med. 2016 Jan 1;2:1–6.
126. Keogh JWL, Power N, Wooller L, Lucas P, Whatman C. Physical and psychosocial function in residential aged-care elders: Effect of Nintendo Wii Sports games. J Aging Phys Act. 2014;22(2):235–44.
127. Adcock M, Thalmann M, Schättin A, Gennaro F, de Bruin ED. A Pilot Study of an In-Home Multicomponent Exergame Training for Older Adults: Feasibility, Usability and Pre-Post Evaluation. Front Aging Neurosci. 2019 Nov 22;11.
128. Jessen JD, Lund HH. Playful home training for falls prevention. In: IEEE/ASME International Conference on Advanced Intelligent Mechatronics, AIM. Institute of Electrical and Electronics Engineers Inc.; 2015. p. 311–7.
129. Saenz-De-Urturi Z, Garcia-Zapirain Soto B. Kinect-based virtual game for the elderly that detects incorrect body postures in real time. Sensors (Switzerland). 2016 May 1;16(5).
130. Vaziri DD, Aal K, Ogonowski C, Von Rekowski T, Kroll M, Marston HR, et al. Exploring user experience and technology acceptance for a fall prevention system: results from a randomized clinical trial and a living lab. Eur Rev Aging Phys Act. 2016 Jun 10;13(1).
131. Vallabhajosula S, McMillion AK, Freund JE. The effects of exergaming and treadmill training on gait, balance, and cognition in a person with Parkinson’s disease: A case study. Physiother Theory Pract. 2017 Dec 2;33(12):920–31.
132. Blair CK, Harding E, Wiggins C, Kang H, Schwartz M, Tarnower A, et al. A home-based mobile health intervention to replace sedentary time with light physical activity in older cancer survivors: Randomized controlled pilot trial. JMIR Cancer. 2021 Apr 1;7(2).
133. Simpson DB, Bird ML, English C, Gall SL, Breslin M, Smith S, et al. “Connecting patients and therapists remotely using technology is feasible and facilitates exercise adherence after stroke.” Top Stroke Rehabil. 2020 Feb 17;27(2):93–102.
134. Fu AS, Gao KL, Tung AK, Tsang WW, Kwan MM. Effectiveness of Exergaming Training in Reducing Risk and Incidence of Falls in Frail Older Adults with a History of Falls. Arch Phys Med Rehabil. 2015 Dec 1;96(12):2096–102.
135. Barisch-Fritz B, Bezold J, Scharpf A, Trautwein S, Krell-Roesch J, Woll A. ICT-Based Individualized Training of Institutionalized Individuals With Dementia. Evaluation of Usability and Trends Toward the Effectiveness of the InCoPE-App. Front Physiol. 2022 Jul 8;13.
136. Park J, Heilman KJ, Sullivan M, Surage J, Levine H, Hung L, et al. Remotely supervised home-based online chair yoga intervention for older adults with dementia: Feasibility study. Complement Ther Clin Pract [Internet]. 2022 Aug;48:101617. Available from: https://linkinghub.elsevier.com/retrieve/pii/S1744388122000858
137. Uzor S, Baillie L. Investigating the long-term use of exergames in the home with elderly fallers. In: Conference on Human Factors in Computing Systems - Proceedings. Association for Computing Machinery; 2014. p. 2813–22.
138. Delbaere K, Valenzuela T, Lord SR, Clemson L, Zijlstra GAR, Close JCT, et al. E-health StandingTall balance exercise for fall prevention in older people: Results of a two year randomised controlled trial. BMJ. 2021 Apr 6;373.
139. Lauzé M, Martel DD, Aubertin-Leheudre M. Feasibility and Effects of a Physical Activity Program Using Gerontechnology in Assisted Living Communities for Older Adults. J Am Med Dir Assoc. 2017 Dec 1;18(12):1069–75.
140. Bao T, Carender WJ, Kinnaird C, Barone VJ, Peethambaran G, Whitney SL, et al. Effects of long-term balance training with vibrotactile sensory augmentation among community-dwelling healthy older adults: A randomized preliminary study. J Neuroeng Rehabil. 2018 Jan 18;15(5):1–13.
141. Hsu YI, Chen YC, Lee CL, Chang NJ. Effects of diet control and telemedicine-based resistance exercise intervention on patients with obesity and knee osteoarthritis: A randomized control trial. Int J Environ Res Public Health. 2021 Aug 1;18(15).
142. Middleton A, Simpson KN, Bettger JP, Bowden MG. COVID-19 Pandemic and Beyond: Considerations and Costs of Telehealth Exercise Programs for Older Adults With Functional Impairments Living at Home—Lessons Learned From a Pilot Case Study. Phys Ther [Internet]. 2020 Aug 12;100(8):1278–88. Available from: https://academic.oup.com/ptj
143. Studenski S, Perera S, Hile E, Keller V, Spadola-Bogard J, Garcia J. Interactive video dance games for healthy older adults. J Nutr Heal aging [Internet]. 2010 Dec;14(10):850–2. Available from: https://linkinghub.elsevier.com/retrieve/pii/S1279770723024363
144. Radhakrishnan K, Julien C, O’Hair M, Baranowski T, Lee G, Allen C, et al. Usability Testing of a Sensor-Controlled Digital Game to Engage Older Adults with Heart Failure in Physical Activity and Weight Monitoring. Appl Clin Inform. 2020 Oct 1;11(5):873–81.
145. Adcock M, Fankhauser M, Post J, Lutz K, Zizlsperger L, Luft AR, et al. Effects of an In-home Multicomponent Exergame Training on Physical Functions, Cognition, and Brain Volume of Older Adults: A Randomized Controlled Trial. Front Med [Internet]. 2020 Jan 28;6. Available from: https://www.frontiersin.org/article/10.3389/fmed.2019.00321/full
146. Jung Y, Li KJ, Janissa NS, Gladys WLC, Lee KM. Games for a better life: effects of playing Wii games on the well-being of seniors in a long-term care facility. In: Proceedings of the Sixth Australasian Conference on Interactive Entertainment [Internet]. New York, NY, USA: ACM; 2009. p. 1–6. Available from: https://dl.acm.org/doi/10.1145/1746050.1746055
147. Khor KX, Chern PM, Yeong CF, Su ELM, Mustar MF, Abdullah N Bin, et al. Smart Balance Board to Improve Balance and Reduce Fall Risk: Pilot Study. In 2018. p. 35–9. Available from: http://link.springer.com/10.1007/978-981-10-7554-4_6
148. Smith ST, Davies TA, Lennox J. Step Training System: An ICT solution to measure and reduce fall risk in older adults. In: 2013 35th Annual International Conference of the IEEE Engineering in Medicine and Biology Society (EMBC) [Internet]. IEEE; 2013. p. 7033–5. Available from: http://ieeexplore.ieee.org/document/6611177/
149. Weinstock RS, Brooks G, Palmas W, Morin PC, Teresi JA, Eimicke JP, et al. Lessened decline in physical activity and impairment of older adults with diabetes with telemedicine and pedometer use: results from the IDEATel study. Age Ageing [Internet]. 2011 Jan;40(1):98–105. Available from: https://academic.oup.com/ageing/article-lookup/doi/10.1093/ageing/afq147
